# Left ventricular unloading in patients with cardiogenic shock treated with veno-arterial extracorporeal membrane oxygenation

**DOI:** 10.1093/ehjopen/oeaf103

**Published:** 2025-08-21

**Authors:** Bilaal Yousaf Dar, Gaayen Ravii Sahgal, Tavgah Jafar, Sangwoo R Jung, Mahmood Ahmad, Rui Bebiano Da Providencia E. Costa, Iqra Javid, Syed Yousaf Ahmad, Malik Takreem Ahmad, Yusuf Abdirahman Yusuf, Abdulrahman Kashkosh

**Affiliations:** Faculty of Life Sciences and Medicine, King’s College London, Guy's Campus, Great Maze Pond, London SE1 1UL, UK; Faculty of Life Sciences and Medicine, King’s College London, Guy's Campus, Great Maze Pond, London SE1 1UL, UK; Faculty of Life Sciences and Medicine, King’s College London, Guy's Campus, Great Maze Pond, London SE1 1UL, UK; Department of Psychiatry, University of Cambridge, Herchel Smith Building, Forvie Site, Robinson Way, Cambridge CB2 0SZ, UK; Royal Free Hospital, Tahir Heart Institute, Pond St, London NW3 2QG, UK; Institute of Health Informatics, University College London, 222 Euston Rd., London NW1 2DA, UK; Faculty of Life Sciences and Medicine, King’s College London, Guy's Campus, Great Maze Pond, London SE1 1UL, UK; St. George’s University Hospital, Blackshaw Rd, London SW17 0QT, UK; Faculty of Life Sciences and Medicine, King’s College London, Guy's Campus, Great Maze Pond, London SE1 1UL, UK; Faculty of Life Sciences and Medicine, King’s College London, Guy's Campus, Great Maze Pond, London SE1 1UL, UK; Calderdale and Huddersfield NHS Foundation Trust, Acre St, Lindley, Huddersfield HD3 3EA, UK

**Keywords:** Impella, Intra-Aortic Balloon Pump (IABP), Left ventricular unloading, Meta-analysis, Veno-Arterial Extracorporeal Membrane Oxygenation (VA-ECMO)

## Abstract

**Aims:**

Cardiogenic shock remains a significant cause of mortality despite multiple advancements in medical interventions. Veno-arterial extracorporeal membrane oxygenation (VA-ECMO) provides crucial circulatory support but also increases left ventricular (LV) after-load, potentially worsening outcomes. Effective LV unloading strategies can enhance patient survival during VA-ECMO treatment. Our aim was to evaluate the impact of LV unloading strategies, including intra-aortic balloon pump (IABP) and Impella, on outcomes such as mortality and adverse effects in patients with cardiogenic shock treated with VA-ECMO.

**Methods and results:**

A systematic search of EMBASE and Medline was conducted from inception up to 20 August 2024. Additional sources included forward citation searches of primary references. Inclusion criteria were studies reporting mortality rates in patients undergoing VA-ECMO with and without LV unloading. Exclusion criteria included case studies, editorials, commentaries, literature reviews, studies without a control group, those not examining LV unloading, studies on non-cardiogenic shock patients, and paediatric populations. From 943 identified studies, 26 met the inclusion criteria after abstract and full text screening by two authors. Data extraction followed PRISMA guidelines with independent reviewers abstracting data and assessing study quality using the Cochrane Risk of Bias in non-randomized studies (ROBINS-I) tool. A random-effects model was used to pool data, accounting for study heterogeneity. The primary outcome was all-cause mortality, assessed at three time points: intra-hospital mortality, 30-day mortality and mortality at longest available follow-up. Secondary outcomes included adverse effects such as bleeding, infection, cardiovascular events, limb ischaemia, and renal replacement therapy (RRT). The meta-analysis included 26 studies with a total of 22 625 patients. LV unloading strategies significantly reduced mortality compared to no unloading (RR: 0.80; 95% CI: 0.73 to 0.96). IABP (RR: 0.78; 95% CI: 0.69 to 0.89) was associated with a significant reduction of mortality compared to no unloading. All adverse effects were comparable across groups apart from significantly increased infection rates and need for RRT in Impella patients (RR: 1.37; 95% CI: 1.07 to 1.75, and RR: 2.02; 95% CI: 1.37 to 3.00, respectively).

**Conclusion:**

LV unloading strategies associated with reduced mortality in patients with cardiogenic shock treated with VA-ECMO. Whilst adverse effects are similar across all strategies, Impella specifically is linked to higher infection rates and need for RRT. These findings could be used to support the use of LV unloading devices in clinical practice and highlight the need for further randomized controlled trials to establish optimal device-options and management protocols.

## Graphical Abstract

**Graphical Abstract:**
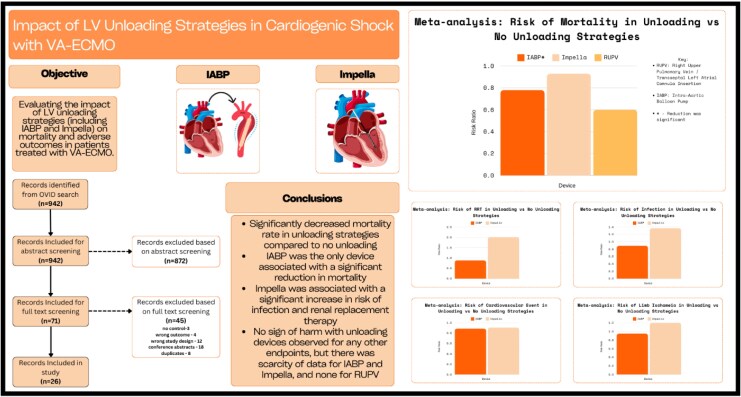

## Introduction

Cardiogenic shock is characterized by inadequate tissue perfusion due to the inability to maintain sufficient cardiac output, which ultimately leads to end-organ hypoperfusion and failure if left untreated.^1,2^ Despite advancements in medical therapy and interventions, mortality rates associated with cardiogenic shock remain high at 59% overall over a 5-year follow-up,^3^ underscoring the need for effective management strategies.

The current management of cardiogenic shock involves a multi-disciplinary approach to optimize cardiac function and restore tissue perfusion.^4–6^ One of the key interventions gaining increasing attention is utilization of veno-arterial extracorporeal membrane oxygenation (VA-ECMO). VA-ECMO can provide temporary circulatory and respiratory support by bypassing the heart and lungs, thereby assisting in oxygenation and maintaining adequate perfusion.^7^ Its role in the management of cardiogenic shock remains controversial. Despite limited evidence supporting its efficacy in improving outcomes^8–10^ it is still considered an option for the management in well-defined cases.^11^

By providing immediate haemodynamic support, VA-ECMO can also stabilize patients in critical condition, allowing time for definitive treatment or recovery.^12^ Additionally, VA-ECMO can facilitate myocardial recovery by reducing overall myocardial oxygen consumption and providing rest to the failing heart.^13^

However, the use of VA-ECMO is not without risks, due to its effects on left ventricular (LV) haemodynamics. Whilst VA-ECMO can alleviate the workload on the heart, it may also lead to adverse effects such as an increased LV after-load, which can exacerbate myocardial injury and impair recovery.^14,15^ Therefore, strategies for unloading the LV are crucial to optimize the benefits of VA-ECMO whilst minimizing potential complications.^16^

Current strategies for unloading the LV can encompass both pre-load and after-load reduction techniques. Pre-load reduction aims to decrease the overall volume of blood returning to the heart, thus reducing LV end-diastolic pressure and myocardial oxygen demand. After-load reduction focuses on decreasing the total resistance against which the LV must pump, thereby easing myocardial workload and improving efficiency.^17^ These unloading strategies include the use of vasodilators, diuretics, intra-aortic balloon pump (IABP), right upper pulmonary vein/trans-septal left atrial cannula (RUPVTLAC), and ventricular assist devices such as Impella.^18,19^

This meta-analysis aimed to comprehensively evaluate the current methods for LV unloading in patients with cardiogenic shock who receive VA-ECMO, and their effects on patient mortality. In addition, we have analysed data on the adverse effects experienced in patients on the unloading strategies. By synthesizing existing evidence from relevant studies, this analysis provides data on optimal management strategies and guides future research directions in the management of cardiogenic shock. According to our knowledge, this is the largest published meta-analysis on LV unloading strategies in VA-ECMO patients to date.

## Methods

### Search strategy

This meta-analysis was performed in accordance with the preferred reporting items for systematic reviews and meta-analyses (PRISMA), the checklist has been reported in Supplementary material online, *Figure S1*.^20^ The protocol was registered on PROSPERO (CRD42024580078). A systematic search of EMBASE and Medline was conducted from inception up until 20 August 2024. Additionally, forward citation searches of the primary reference list were conducted. The search strategy consisted of a combination of keywords related to LV unloading techniques and ECMO, which were devised by two independent reviewers (B.D. and I.J.). The finalized search terms were: ‘Impella’; ‘IABP’; ‘VA-ECMO’; and ‘Transaortic Catheter’. The search strategy was input into Ovid for use and only papers with full-text language in English and human subjects were included (see Supplementary Material—Annex  *A*: Search Strategy). The resulting citations were imported to Rayyan, and duplicates were removed by manual inspection by two authors (B.D. and I.J.).

### Study selection, data extraction, and quality assessment

#### PICO question

In patients treated with VA-ECMO following cardiogenic shock, how do LV unloading strategies such as Impella and IABP compare with no unloading strategies? The primary outcome for this was all-cause mortality, and the secondary outcomes were adverse effects including bleeding events, infection rates, cardiovascular events, limb ischaemia and renal replacement therapy (RRT).

The inclusion criteria were papers using LV unloading strategies such as IABP and Impella, and publishing mortality outcomes. The exclusion criteria were (i) case studies or case reviews, (ii) editorials, (iii) commentaries, (iv) literature or systematic reviews, (v) no control group given, and (vi) papers with patients under 18 years of age. Discrepancies in the results of independent reviews were resolved by consensus.

The primary outcome for the meta-analysis was all-cause mortality. This was assessed at three time points: intra-hospital mortality (IHM), 30-day mortality (from time of index procedure) and mortality at longest available follow-up. Secondary outcomes included adverse effects such as bleeding, infection, cerebrovascular accidents (CVA), limb ischaemia and RRT. Other data extracted included year of data collection, design of study, aetiology, comorbidities, age, sex, BMI, ethnicity, country, previous use of unloading devices, median length of stay, and the time of recording mortality. Wherever possible, propensity matched data was used in an attempt to overcome confounding effects.

### Risk of bias assessment

The included articles were assessed for risk of bias using Cochrane’s Risk of Bias in Non-randomized Studies (ROBINS-I) tool.^21^ The ROBINS-I tool has a total of seven domains of bias: bias due to (i) confounding, (ii) selection of participants, (iii) classification of interventions, (iv) deviations from intended interventions, (v) missing data, (vi) measurement of outcomes, and (vii) selection of reported result. By individually judging a paper’s bias in each domain as ‘low’, ‘moderate’, ‘serious’, or ‘critical’, an overall risk of bias judgement was reached by using the most severe judgement for that paper in-line with Cochrane’s latest guidance on using ROBINS-I. Two authors independently (B.D. and G.S.) assessed each paper and discussed discrepancies to reach a judgement. If they were unable to come to a consensus, a third author was consulted to resolve disagreements.

### Statistical analysis

Univariate risk ratios (RRs) were calculated using the Paul–Mandel random-effects method, which allowed for heterogeneity in effect sizes between studies. This was used for both the primary and secondary outcomes. Heterogeneity was assessed using the I^2^ statistic. Publication bias was determined using Egger regression and visual inspection of funnel plots. Two-sided *P* values of <0.05 were considered significant. We utilized R and the 786-MIII RR/odds ratio Forest-plot meta-analysis programme.^22^

Numbers needed to treat (NNT) were presented for endpoints where interventions showed a significant benefit or harm.

## Results

### Literature search

**Figure 1:**
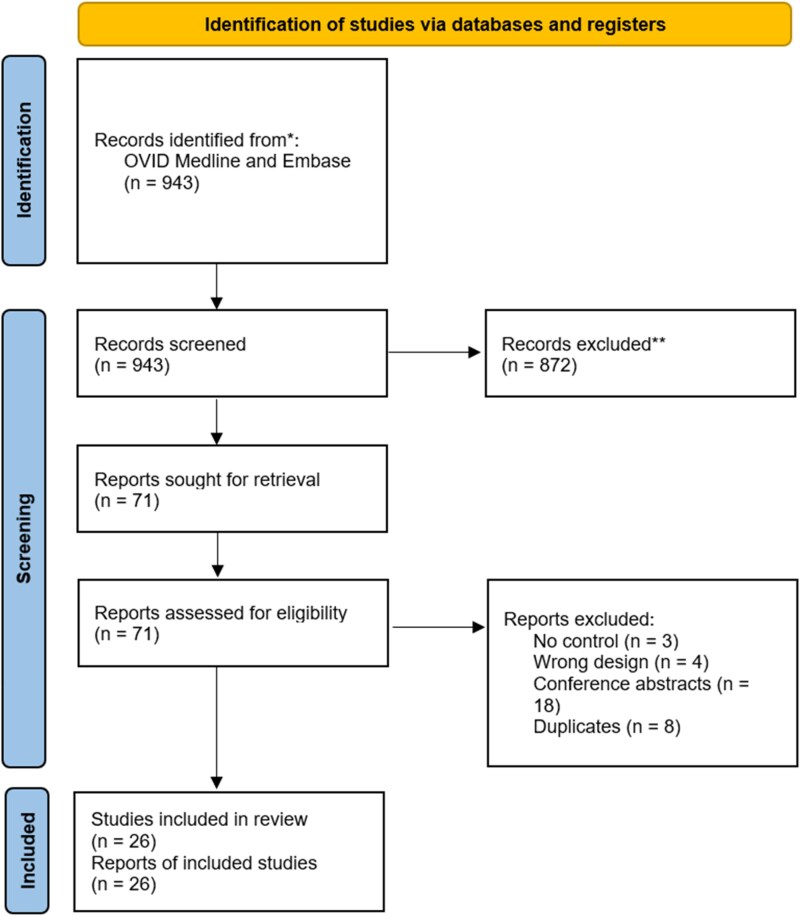
PRISMA flowchart with study selection.

Our initial literature search retrieved 943 records. These were imported into Rayyan for screening by two independent reviewers (B.D. and I.J.) to identify potentially eligible studies. After title and abstract screening, 71 studies were deemed of potential interest and the full text was revised. 45 studies were removed for the following reasons: no control (3), wrong outcome (4), wrong study design (12), conference abstracts (18), and duplicate (8) (see Supplementary material online, *Table S1*). 26 studies^23–48^ were included in this meta-analysis (*Figure 1*). All the studies had a retrospective, observational design, save two which were prospective.

**Figure 2:**
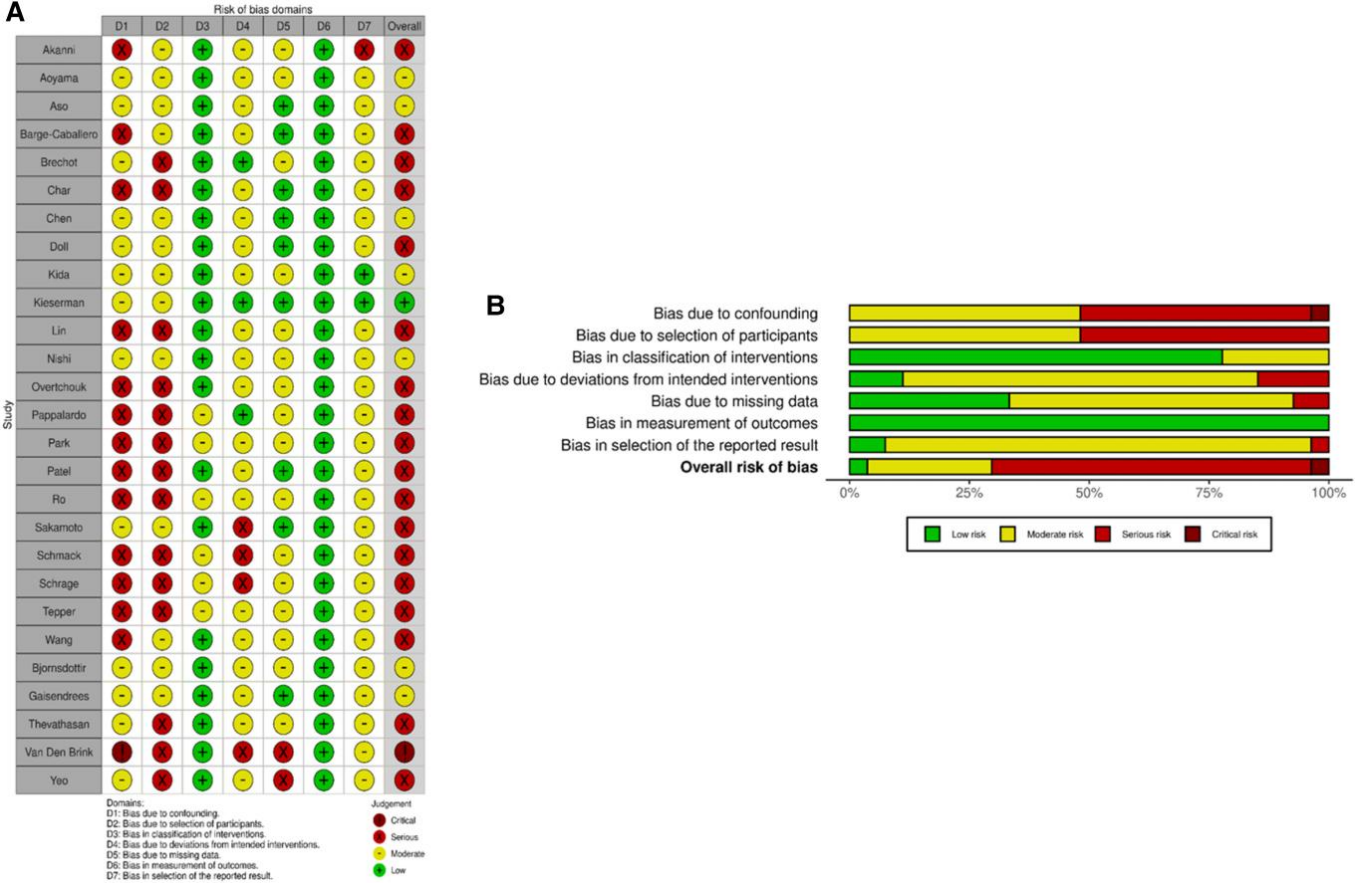
A traffic light chart showing the risk of bias judgements for the seven domains and overall risk of bias using the ROBINS-I assessment (*A*), and bar chart overview of the results of the risk of bias assessment using ROBINS-I (*B*).

Five domains had studies with serious risk of bias (e.g. bias due to confounding, selection of participants, deviations from intended interventions, missing data, and selection in reported results). Overall, more than half of studies (*n* = 18) were considered to have serious risk, and one study had critical risk. Only one study was considered low risk. The summary of ROBINS-I judgements is presented in *Figure 2*.

A total population of 22 581 patients were included in this review. A summary of the baseline characteristics can be seen in *Table 1* below.

**Table 1:** Baseline characteristics

Study	Design	Arm	Patients	Aetiology	Comorbidities	Age	Male %	BMI Mean
**Akanni** (2010–2014)	Retrospective observational, SC	Impella	14	Post-cardiotomy shock, acute MI, primary graft failure, acute decompensated HF	CAD, HL, HTN, COPD, DM, Prev CVA, CKD	63^a^	78.57	30.86
		No unloading	196			57^a^	67.86	28.55
**Aoyama** (2014)^24^	Retrospective observational, SC	IABP	35	–	–	–	–	–
		No unloading	3			–	–	–
**Aso** (2010–2013)	Retrospective observational, MC	IABP	533	IHD, HF, carditis, cardiomyopathy, valvular disease, IE	–	60–79^a^	71.9	–
		No Unloading	533			60–79^a^	73.4	–
**Bjornsdottir** (2010–2018)	Retrospective observational, MC	IABP	114	HF, cardiac arrest, vent arrhythmia, resp arrest, septic shock	DM, renal failure, aortic dissection, AF, stroke	62^b^	70	27
		No Unloading	114			62^b^	70	27
**Brechot** (2007–2012)	Retrospective observational, SC	IABP	63	Refractory shock, cardiac arrest	–	52^a^	79.4	25^a^
		No unloading	63			53^a^	69.8	26^a^
**Char** (2015–2020)	Retrospective observational, SC	IABP	68	Acute MI, decompensated HF, vent arrhythmia, myocarditis, PE	CAD, CHF, HL, DM, HTN, COPD, prev CVA, CKD	59.5^a^	69.1	–
		Impella	72			62^a^	70.8	–
		No Unloading	143			58^a^	58	–
**Doll** (1997–2002)	Prospective observational, SC	IABP	143	–	DM, HTN, HL	61.3^b^	73	27
		No unloading	76					
**Gaisendrees** (2016–2020)	Retrospective observational, SC	Impella	18	MI, arrhythmia, PE, myocarditis	–	57^a^	84	–
		No unloading	90			56.5^a^	82	–
**Kai Chen** (2005–2017)	Retrospective observational, SC	IABP	38	–	HTN, HL, DM, AF, smoking	49.5^b^	73.7	23.6
		No unloading	22					
**Kida** (1998–2014)	Prospective observational, MC	IABP	459	–	HTN, DM, HL, smoking, prev STEMI	66.35^b^	80.1	24.09
		No unloading	60			70.84^b^	66.1	22.86
**Kieserman** (2017–2022)	Retrospective observational, SC	Impella	16	Acute MI	CAD, Prev CABG, HTN, smoking	59^b^	81.2	–
		No unloading	34			55.5^b^	79.4	–
**Lin** (2002–2013)	Retrospective observational, SC	IABP	302	ACS, cardiomyopathy, acute myocarditis	HTN, DM, CKD, liver cirrhosis, COPD, CAD, Prev MI, stroke	56.8^b^	79.5	25.1
		No unloading	227			52.8^b^	70	23.9
**Nishi** (2012–2018)	Retrospective observational, MC	IABP	846	–	HTN, HL, AF, DM, renal, malignancy	69^a^	77.1	–
		No unloading	846			69^a^	78.5	–
**Overtchouk** (2007–2013)	Retrospective observational, SC	IABP	63	–	MI, CAD, Prev CABG, smoking, HTN, DM, dyslipidaemia	52.7^b^	84	–
		No unloading	43					
**Pappalardo** (2015–2015)	Retrospective observational, MC	Impella	34	–	–	54^a^	82	–
		No unloading	123			55^a^	83	–
**Park** (2004–2011)	Retrospective observational, SC	IABP	41	–	DM, HTN, HL, Prev MI, Prev PCI, smoking	66^a^	75.6	24^a^
		No unloading	55			64^a^	78.2	22.8^a^
**Patel** (2014–2016)	Retrospective observational, SC	Impella	30	–	–	55^a^	67	32^a^
		No Unloading	36			63^a^	70	33^a^
**Ro** (2005–2012)	Retrospective observational, SC	IABP	60	–	DM, HTN, renal failure, liver cirrhosis	64.1^b^	71.7	24.1
		No unloading	193			57.1^b^	57.5	23.3
**Sakamoto** (2000–2010)	Retrospective observational, SC	IABP	94	Cardiogenic shock, acute MI, cardiac arrest	HTN, DM, stroke	72^b^	66.3	–
		No unloading	4					
**Schrage** (2005–2019)	Retrospective observational, SC	Impella	255	Acute MI, other	–	56.05^b^	64.4	–
		No unloading	255			57.56^b^	62.6	–
**Shmack** (2004–2014)	Retrospective observational, SC	RUPV or TLAC insertion	20	DCM, acute myocarditis, MI, ischaemic cardiomyopathy	–	38.3^b^	70	23.1
		No unloading	28			57.9^b^	50	27.2
**Tepper** (2010–2016)	Retrospective observational, SC	IABP	30	Acute MI, ischaemic cardiomyopathy, myocarditis, non-ischaemic cardiomyopathy, post-cardiotomy shock, graft dysfunction, other	CAD, cardiomyopathy, Prev MI, HTN, DM, HL, stroke, renal failure	57.2^b^	60	–
		No unloading	30			50.5^b^	47	–
**Thevathasan** (2016–2021)	Retrospective observational, MC	Impella	34	–	Acute MI, cardiac arrest, HL, DM, HTN, smoking, obesity	65.2^b^	79.4	27.1
		No unloading	34			67.1^b^	79.4	27.3
**Van den Brink** (2015–2018)	Retrospective observational, MC	IABP	7	–	–	59^a^	–	–
		No unloading	11			59^a^	–	–
**Wang** (2004–2011)	Retrospective observational, SC	IABP	41	Rheumatic disease, degenerative disease, congenital disease, ischaemia	AF, HTN, DM, renal failure, stroke	65^b^	64	–
		No unloading	46					
**Yeo** (2016–2020)	Retrospective observational, MC	No unloading	10 262	MI	Prev cardiac arrest, HTN, DM, anaemia, CKD, AF, COPD, CHF	55^a^	63.2	–
		IABP	3098			59^a^	67.5	–
		Impella	2620			58^a^	74.8	–

ACS, acute coronary syndrome; AF, atrial fibrillation; CABG, coronary artery bypass grafting; CHF, congestive heart failure; CKD, chronic kidney disease; COPD, chronic obstructive pulmonary disease; CVA, cerebrovascular accident; DCM, dilated cardiomyopathy; DM, diabetes mellitus; HL, hyperlipidaemia; HTN, hypertension; IE, infective endocarditis; IHD, ischaemic heart disease; MC, multi-centre study; MI, myocardial infarction; PCI, percutaneous coronary intervention; PE, pulmonary embolism; Prev, previous; RUPV, right upper pulmonary vein; SC, single centre study; STEMI, ST-segment elevation myocardial infarction; TLAC, trans-septal left atrial cannula.

^a^Mean.

^b^Median.

(Year)—Data collection time period.

The population size of the studies ranged from 18 to 15 980. The aetiology of participants varied greatly and included ischaemic heart disease, cardiomyopathy, myocarditism, and valvular disease, hypertension, diabetes mellitus, and hyperlipidaemia were among the most commonly occurring comorbidities.

No studies reported on ethnicity or included data on previous use of unloading devices. Further information collected on the studies can be seen in Supplementary material online, Table  *S*.2.

### Intra-hospital mortality

IHM for the overall cohort was 48% (*n* = 10 088). Both Impella (54% vs. 51%, RR: 0.82; 95% CI: 0.67 to 1.01; eight studies; I^2^ = 80%) and IABP (43% vs. 48%, RR: 0.85; 95% CI: 0.63 to 1.06; 15 studies; I^2^ = 83%) showed comparable results vs. no unloading strategies. Overall unloading strategies showed significantly lower rates of IHM than no unloading strategies. (47% vs. 49%, RR: 0.82; 95% CI: 0.70 to 0.96; 21 studies; I^2^ = 83%) (see Supplementary material online, *Figure S3*).

### 30-Day Mortality

30-Day mortality for the overall cohort was 56% (*n* = 1409). Whilst Impella (57% vs. 59%, RR: 0.89; 95% CI: 0.78 to 1.02; three studies; I^2^ = 0%) was comparable with no unloading, IABP (51% vs. 61%, RR: 0.79; 95% CI: 0.72 to 0.87; four studies; I^2^ = 0%) showed a significant reduction in mortality. Overall unloading strategies showed significantly lower rates of 30-day mortality than no unloading strategies. (52% vs. 60%, RR: 0.82; 95% CI: 0.75 to 0.89; seven studies; I^2^ = 14%) (see Supplementary material online, *Figure S4*).

### Mortality at longest available follow-up

Over a median follow-up of 30 days (range: 10–510, IQR: 16–365), the overall mortality for the entire cohort was 50% (*n* = 11 173). Use of Impella vs. no unloading was not associated with a mortality benefit: 56% vs. 52%, RR: 0.93; 95% CI: 0.78 to 1.11; nine studies; I^2^ = 77%. IABP was associated with significantly lower patient mortality (45% vs. 49%, RR: 0.78; 95% CI: 0.69 to 0.89; 18 studies; I^2^ = 77%; NNT: 25), and trend for potentially lower mortality was observed for RUPVTLAC (45% vs. 75%, RR: 0.60; 95% CI: 0.35 to 1.02; two studies; I^2^ = 82%). Among studies providing data on the comparison of IABP against Impella, there was no significant difference between the two unloading strategies (48% vs. 55%, RR: 0.75; 95% CI: 0.52 to 1.09; two studies; I^2^ = 82%).

**Figure 3:**
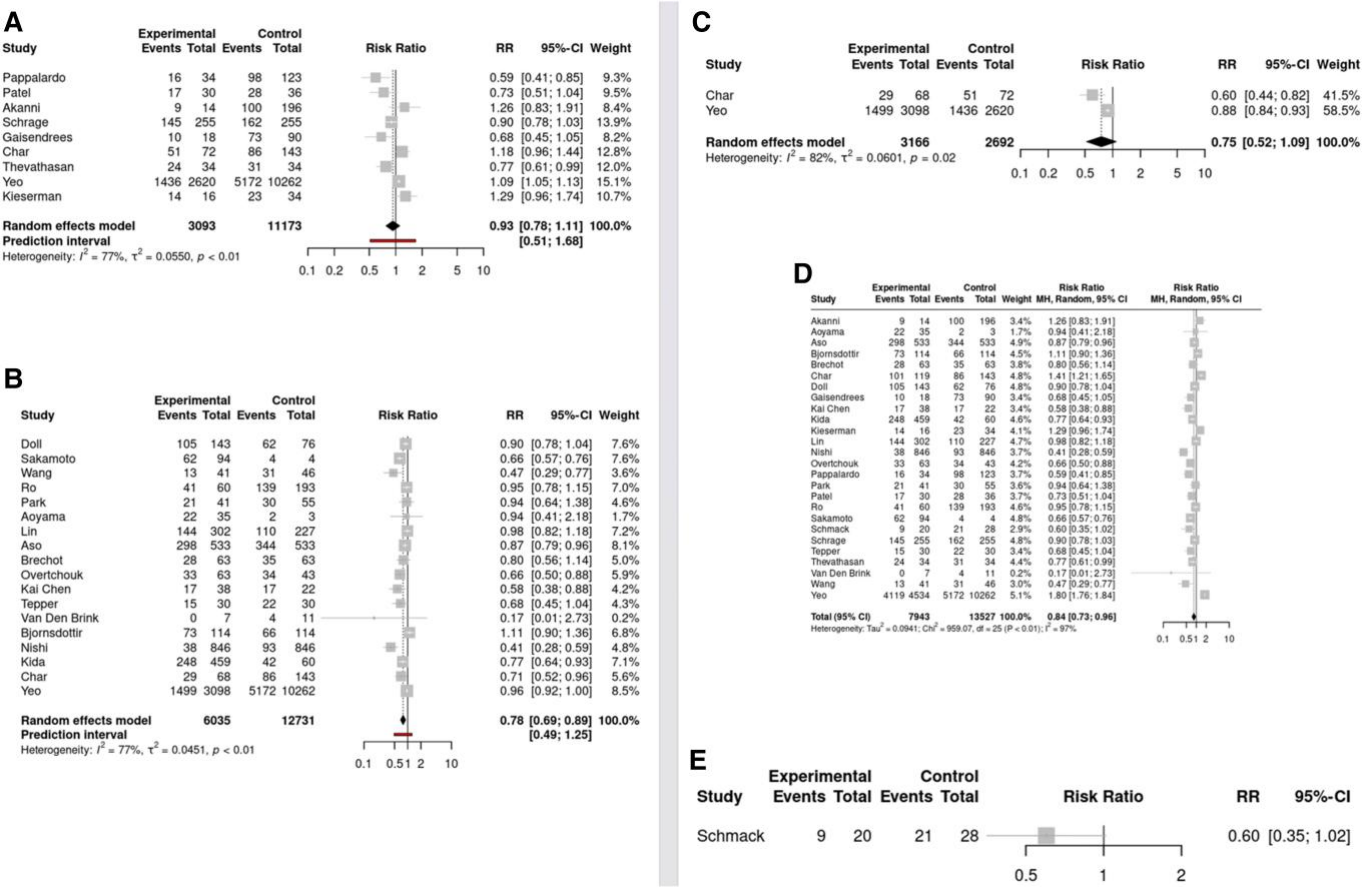
Forest-plots for the outcome all-cause mortality: (*A*) Impella compared to no unloading; (*B*) IABP compared to no unloading; (*C*) IABP compared to Impella; (*D*) overall unloading compared to no unloading; and (*E*) RUPV/TLAC compared to no unloading.

Overall, when pooling all available data, unloading strategies were associated with a significant reduction in mortality when compared to patients with no unloading strategy (50% vs. 71%, RR: 0.84; 95% CI: 0.73 to 0.96; NNT: 4.76; 26 studies; I^2^ = 97%) (*Figure 3*). The funnel-plot suggested publication bias (see Supplementary material online, *Figure S2*).

### Bleeding

Among the 20 480 patients with data on bleeding, the overall event rate was 32% (*n* = 6596.2). Impella (40% vs. 43% RR: 0.95; 95% CI: 0.65 to 1.38; nine studies; I^2^ = 97%) and IABP (27% vs. 32%, RR: 1.00; 95% CI: 0.80 to 1.24; nine studies; I^2^ = 19%) had comparable rates of bleeding when compared to no unloading strategy. No bleeding data was available for RUPVTLAC. When comparing IABP against Impella, there was no significant difference (35% vs. 40%, RR: 0.57; 95% CI: 0.0 to 264,08; two studies; I^2^ = 91%). Overall, the results were comparable between unloading and no unloading strategies (32% vs. 32%, RR: 1.1; 95% CI: 0.91 to 1.32; 16 studies; I^2^ = 74%) (see Supplementary material online, *Figure S5*).

### Infection

**Figure 4:**
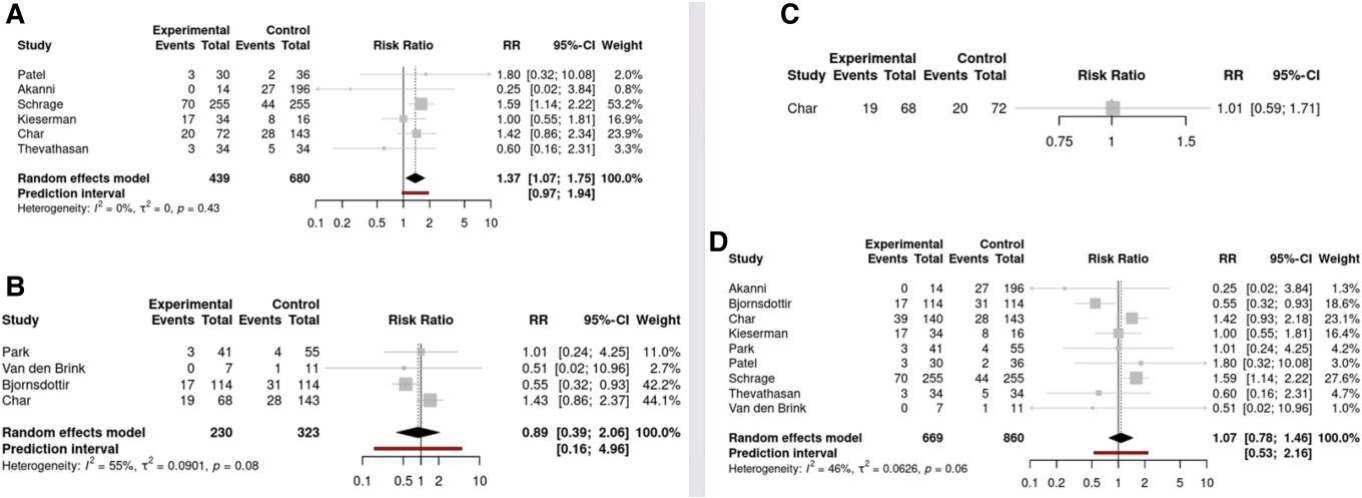
Forest-plots for the outcome infection: (*A*) Impella compared to no unloading; (*B*) IABP compared to no unloading; (*C*) IABP compared to Impella; and (*D*) overall unloading vs. no unloading.

The overall event rate among the 1529 patients with data on infection was 25% (*n* = 388). Impella (26% vs. 17%, RR: 1.37; 95% CI: 1.07 to 1.75; six studies; I^2^ = 0%; NNT: −11.1) was associated with an increased risk of infection when compared to no unloading strategy. On the other hand, infection rate with IABP was comparable to no unloading strategy (20% vs. 17%, RR: 0.89; 95% CI: 0.39 to 2.06; four studies; I^2^ = 55%) (*Figure 4*). No infection data were available for RUPVTLAC. When comparing IABP against Impella, there was no significant difference (28% vs. 28%, RR: 1.01; 95% CI: 0.59 to 1.71; one study). Overall, the results were comparable between unloading and no unloading strategies (23% vs. 17%, RR: 1.07; 95% CI: 0.78 to 1.46; seven studies; I^2^ = 46%) (*Figure 4*).

### Cerebrovascular accident

Among 20 105 patients with data on CVA, the overall event rate was 10% (*n* = 1976). Both IABP (12% vs. 9%, RR: 0.89; 95% CI: 0.71 to 1.10; seven studies; I^2^ = 52%) and Impella (8% vs. 9%, RR: 0.91; 95% CI: 0.8 to 1.04; eight studies; I^2^ = 0%) had comparable CVA event rate to not using unloading devices. No CVA data was available for RUPVTLAC. When comparing IABP against Impella, there was no significant difference (10% vs. 9%, RR: 1.16; 95% CI: 0.99 to 1.37; two studies; I^2^ = 0%). Overall, the results were comparable between unloading and no unloading strategies (11% vs. 9%, RR: 0.88; 95% CI: 0.77 to 1.01; 12 studies; I^2^ = 24%) (see Supplementary material online, *Figure S6*).

### Limb ischaemia (LI)

The overall rate of LI among the 16 755 patients with data on LI was 4% (*n* = 734). Impella (5% vs. 4%, RR: 1.20; 95% CI: 0.89 to 1.62; four studies; I^2^ = 0%) and IABP (4% vs. 4%, RR: 0.95; 95% CI: 0.76 to 1.20; five studies; I^2^ = 0%) showed a comparable risk of LI to no unloading strategy. No LI data was available for RUPVTLAC. When comparing IABP against Impella, there was no significant difference (4% vs. 5%, RR: 0.82; 95% CI: 0.25 to 2.71; two studies; I^2^ = 0%). Overall, the results were comparable between unloading and no unloading strategies (4% vs. 4%, RR: 1.08; 95% CI: 0.93 to 1.26; seven studies; I^2^ = 1%) (see Supplementary material online, *Figure S7*).

### Renal replacement therapy

Among the 20 883 patients with data on RRT, the overall event rate was 16% (*n* = 3241.2). Use of Impella (26% vs. 17% RR: 2.02; 95% CI: 1.37 to 3; six studies; I^2^ = 79%) was associated with a significantly increased risk of RRT vs. no unloading. IABP (9% vs. 16%, RR: 0.89; 95% CI: 0.70 to 1.12; four studies; I^2^ = 28%) had a comparable rate of RRT when compared to no unloading strategy. No RRT data were available for RUPVTLAC.

Overall, the results were comparable between unloading and no unloading strategies (15% vs. 16%, RR: 1.43; 95% CI: 0.88 to 2.34; nine studies; I^2^ = 84%) (see Supplementary material online, *Figure S8*).

### Sensitivity analysis

We tested sensitivity for mortality, infection, and bleeding, in order to determine if any significant changes would occur to the Forest-plots if we were to remove results lying on the extremities of the data range. When analysing mortality, we removed Pappalardo from the Impella analysis, which did not result in any significant change. For IABP mortality, we removed Van Den Brink, which did not result in any significant change. In the infection category, we removed Van Den Brink, which did not result in any significant change. In the Impella bleeding group, we also removed Van Den Brink, which did not result in any significant change.

## Discussion

In this meta-analysis, we compared the use of different LV unloading strategies to combat the increase in LV after-load following ECMO treatment for cardiogenic shock. Overall, we found that unloading strategies associated with reduced mortality rates when compared to no unloading strategy. Of all three assessed strategies (IABP, Impella, and RUPVTLAC), only IABP was associated with a mortality reduction when assessed separately. Adverse effects, such as bleeding, infection, cardiovascular events, and limb ischaemia were common. Use of Impella was associated with a significantly higher infection rate and need for RRT. No sign of harm with unloading devices was observed for any of the other endpoints. There was scarcity of data for IABP and Impella, and no data for RUPV for the assessed secondary endpoints.

### Mortality

Our findings for mortality serve to further strengthen the current body of evidence suggesting that unloading strategies may reduce mortality in VA-ECMO patients. Russo *et al*.^49^ showed similar results in their own meta-analysis to examine the efficacy and safety of LV unloading strategies during ECMO treatment. Additionally, they shared our observation that IABP was associated with the most significant reduction in mortality rates among all the unloading strategies.

High heterogeneity in our mortality analyses (I² = 97%) should be taken into account as it indicates that the variation in effect sizes across studies is not simply due to chance but likely reflects substantial differences in clinical and methodological factors such as patient populations, regional practice patterns, and timing of intervention. This undermines the reliability and generalizability of the pooled estimate, as the summary RR may not accurately represent the effect in any specific context. High heterogeneity can arise from clinical diversity (e.g. differences in shock aetiology, comorbidities, or device selection protocols) and methodological diversity (e.g. variations in study design, outcome definitions, and risk of bias). As a result, the pooled effect may mask important subgroup differences and obscure true treatment effects, limiting its utility for guiding clinical practice. Furthermore, high heterogeneity increases statistical uncertainty, meaning that the range of plausible effects in a new population (as reflected by prediction intervals) may be wide, encompassing both benefit and harm. Therefore, whilst our meta-analysis suggests a mortality benefit with LV unloading, the high heterogeneity necessitates cautious interpretation and highlights the need for further research to identify patient and procedural factors that modify treatment effect.

Whilst our meta-analysis and Russo *et al*.’s found Impella to be crossing the line of null effect, other studies looking into Impella alone have shown different results. Thevathasan *et al*.^50^ reported a significant difference in the mortality between the Impella group and the no unloading group. Whilst this difference could arise due to a multitude of factors, we believe it is likely due to Thevathasan *et al*. focusing on in-hospital mortality only. As Abusnina *et al*.^51^ have shown in their meta-analysis, Impella mortality rates tend to worsen over time and are best in-hospital.

### Adverse effects

Our findings indicate that, with the exception of higher infection rates and need for RRT in patients treated with Impella, unloading strategies conferred similar rates of adverse events to patients as no unloading strategy did. However, numbers of patients for these analyses were low, and it is possible that these were underpowered for showing any clinically significant differences.

Potential mechanisms of harm during RRT can include complications related to catheter size, haemolysis, and infection risk. Large-bore catheters can cause mechanical stress on erythrocytes, leading to haemolysis and subsequent haemoglobinuria, which may contribute to acute kidney injury due to tubular damage from iron deposition and oxidative stress.^52^ This haemolytic risk is exacerbated when RRT is combined with other extracorporeal circuits like ECMO, where turbulence and high shear forces further damage red blood cells.^53^ Additionally, large cannula size increases the risk of catheter-related bloodstream infections due to greater endothelial disruption and biofilm formation.^54^ Sepsis risk is amplified by immune dysfunction in acute kidney injury patients, who exhibit impaired pathogen clearance and heightened susceptibility to severe infections.^55^

Across all studied adverse effects, Impella was associated with numerically, but not significantly, higher rates of adverse events than IABP, especially in bleeding events. Gandhi *et al*. suggest that the increased bleeding rates may be due to the larger sheaths used in Impella (12F-14F) as compared to those used in IABP (7F-8F), as well as the angulation required when inserting the Impella device.^56^ Further studies suggest increased rates of major bleeding events linked with Impella use but not IABP.^57,58^

### Current indications for impella and IABP

Recent randomized and observational studies have clarified the indications and comparative outcomes of Impella and IABP for LV unloading in patients with cardiogenic shock, particularly those supported with VA-ECMO. Whilst both devices are used to mitigate the adverse effects of increased LV after-load during mechanical circulatory support, evidence from large meta-analyses and registry studies indicates that IABP is associated with a significant reduction in mortality compared to no unloading, and may offer a safer profile with lower rates of bleeding and acute kidney injury compared to Impella.^48^ In contrast, Impella use has not consistently demonstrated a mortality benefit over IABP and is linked to higher risks of major bleeding, haemolysis, infection, and need for RRT.^48^ These findings suggest that, whilst both Impella and IABP are indicated for LV unloading in refractory cardiogenic shock, IABP may be preferred in many cases due to its favourable safety and efficacy profile, with Impella reserved for selected patients where greater unloading is required and the benefits outweigh the risks.^56^ Further randomized trials are needed to refine patient selection and optimize device choice in this high-risk population.

### Study limitations

All the studies included in our meta-analysis were retrospective observational studies, apart from two which were prospective observational.^29,32^ Quality of most studies was low. Accordingly, our results may be subject to bias from confounding variables which we were unable to control for. In an attempt to overcome this, we used propensity-matched data whenever possible. Major factors that we were unable to control for include background therapy and comorbidities. Length of stay was also sparsely available, which could have impacted results. Moreover, whilst some studies reported the timing of unloading device initiation relative to ECMO, data was insufficiently detailed or inconsistently stratified to allow for formal comparisons between patients who received unloading before, concurrently with, or after ECMO initiation—a variable that may have confounded outcome analyses. Where available, timing data were recorded (see Supplementary material online, *Table S2*). Despite including studies from all across the globe, data on ethnicity was not reported in any studies, which means our results may not be generalizable to all ethnicities and populations (see Supplementary material online, *Table S2*). Additionally, data was frequently unavailable for our secondary analysis on rates of adverse effects, potentially leading to possible bias in the data. High heterogeneity was observed for most of the assessed outcomes.

### Future trials

We suggest future research should focus on randomized controlled trials for the efficacy and safety of unloading strategies against no unloading strategy. We believe it is important for any such future study to control background therapies and comorbidities, whilst acknowledging this is not an easy task. Such trials should compare the use of different unloading strategies to devise gold-standard clinical practice and for use as the basis of guidelines for LV unloading after cardiogenic shock.

## Conclusion

Unloading strategies were associated with a lower mortality rate when compared to no unloading strategy in patients on VA-ECMO for cardiogenic shock. Except for higher infection rate and need for RRT in patients treated with Impella, adverse effects were comparable across patients receiving and not receiving LV unloading devices. Future randomized controlled trials are needed to further explore the best strategy for LV unloading.

## Supplementary Material

oeaf103_Supplementary_Data

## Data Availability

No new data were generated or analysed in support of this research. All data used in this study were derived from previously published sources, which are cited in the references.

## Lead Author Biography

Bilaal Dar is a final-year medical student at King’s College London, passionate about research and surgery. His work has been published in a variety of journals and he has been invited to present his research at the national level. Bilaal is the founder of SharpStart Learning, an innovative educational company supporting school students across the UK. He is also an active member of the national research team AMRA and facilitates access for medical students into research. Recognized by his achievement of the AMA International Award for Academic Excellence, Bilaal’s work displays his commitment to academia and humanitarian service.
