# Supplementary material for: Left ventricular unloading in patients with cardiogenic shock treated with veno-arterial extracorporeal membrane oxygenation
Source: Eur Heart J Open. 2025 Aug 21;5(5):oeaf103. doi: 10.1093/ehjopen/oeaf103 (PMC12415181; doi:10.1093/ehjopen/oeaf103)
Supplement: oeaf103_Supplementary_Data [file oeaf103_supplementary_data.docx]

**Supplementary Material**

**Figure S.1: PRISMA Checklist**

# PRISMA 2020 Main Checklist

| **Topic** | **No.** | **Item** | **Location where item is reported** |
| --- | --- | --- | --- |
| **TITLE** |  |  |  |
| **Title** | 1 | Identify the report as a systematic review. | Title |
| **ABSTRACT** |  |  |  |
| **Abstract** | 2 | See the PRISMA 2020 for Abstracts checklist |  |
| **INTRODUCTION** |  |  |  |
| **Rationale** | 3 | Describe the rationale for the review in the context of existing knowledge. | The rationale is explained in the introduction of the meta-analysis, stating the need for evaluating LV unloading strategies during VA-ECMO due to high mortality rates in cardiogenic shock patients​ |
| **Objectives** | 4 | Provide an explicit statement of the objective(s) or question(s) the review addresses. | The objective is to evaluate the impact of LV unloading strategies (IABP and Impella) on mortality and adverse outcomes in patients with cardiogenic shock |
| **METHODS** |  |  |  |
| **Eligibility criteria** | 5 | Specify the inclusion and exclusion criteria for the review and how studies were grouped for the syntheses. | The inclusion and exclusion criteria are clearly outlined in the methods section. Inclusion criteria involved studies reporting mortality rates in patients undergoing VA-ECMO with and without LV unloading. Exclusion criteria included case studies, editorials, commentaries, and studies without control groups​ |
| **Information sources** | 6 | Specify all databases, registers, websites, organisations, reference lists and other sources searched or consulted to identify studies. Specify the date when each source was last searched or consulted. | The report specifies that a systematic search of EMBASE, Medline, and forward citation searches was conducted. The last search date is also provided (up to August 2024)​ |
| **Search strategy** | 7 | Present the full search strategies for all databases, registers and websites, including any filters and limits used. | The search strategy, including the terms and filters used, is presented in the Supplementary Material​ |
| **Selection process** | 8 | Specify the methods used to decide whether a study met the inclusion criteria of the review, including how many reviewers screened each record and each report retrieved, whether they worked independently, and if applicable, details of automation tools used in the process. | The methods for screening studies, involving two independent reviewers, are described​ |
| **Data collection process** | 9 | Specify the methods used to collect data from reports, including how many reviewers collected data from each report, whether they worked independently, any processes for obtaining or confirming data from study investigators, and if applicable, details of automation tools used in the process. | Data collection followed PRISMA guidelines, and data was collected by two independent reviewers |
| **Data items** | 10a | List and define all outcomes for which data were sought. Specify whether all results that were compatible with each outcome domain in each study were sought (e.g. for all measures, time points, analyses), and if not, the methods used to decide which results to collect. | Data was collected on primary outcomes like all-cause mortality and secondary outcomes such as bleeding, infection, cardiovascular events, limb ischemia, and renal replacement therapy​ |
|  | 10b | List and define all other variables for which data were sought (e.g. participant and intervention characteristics, funding sources). Describe any assumptions made about any missing or unclear information. | Variables like patient demographics, comorbidities, country, and length of stay were also collected​ |
| **Study risk of bias assessment** | 11 | Specify the methods used to assess risk of bias in the included studies, including details of the tool(s) used, how many reviewers assessed each study and whether they worked independently, and if applicable, details of automation tools used in the process. | The ROBINS-I tool was used for bias assessment, and two independent reviewers were involved in the process​ |
| **Effect measures** | 12 | Specify for each outcome the effect measure(s) (e.g. risk ratio, mean difference) used in the synthesis or presentation of results. | The effect measures used in the meta-analysis include risk ratios (RR) with confidence intervals (CI) for both primary and secondary outcomes​ |
| **Synthesis methods** | 13a | Describe the processes used to decide which studies were eligible for each synthesis (e.g. tabulating the study intervention characteristics and comparing against the planned groups for each synthesis (item 5)). | The methods for synthesizing studies include assessing eligibility based on LV unloading strategies and comparing them against no unloading strategies |
|  | 13b | Describe any methods required to prepare the data for presentation or synthesis, such as handling of missing summary statistics, or data conversions. | The methods for handling missing data, particularly the use of propensity matching, are described in the report |
|  | 13c | Describe any methods used to tabulate or visually display results of individual studies and syntheses. | Results were presented using forest plots, funnel plots, and other graphical representations​ |
|  | 13d | Describe any methods used to synthesize results and provide a rationale for the choice(s). If meta-analysis was performed, describe the model(s), method(s) to identify the presence and extent of statistical heterogeneity, and software package(s) used. | The random-effects model was used for synthesizing results due to heterogeneity among the studies​ |
|  | 13e | Describe any methods used to explore possible causes of heterogeneity among study results (e.g. subgroup analysis, meta-regression). | Heterogeneity was assessed using I² statistics. Subgroup analyses were also performed to explore causes |
|  | 13f | Describe any sensitivity analyses conducted to assess robustness of the synthesized results. | Sensitivity analyses were performed by removing outliers and extreme data points from the analysis to ensure robustness​ |
| **Reporting bias assessment** | 14 | Describe any methods used to assess risk of bias due to missing results in a synthesis (arising from reporting biases). | Publication bias was assessed using funnel plots and Egger regression |
| **Certainty assessment** | 15 | Describe any methods used to assess certainty (or confidence) in the body of evidence for an outcome. | Certainty in the evidence was assessed through sensitivity analyses and consistency checks |
| **RESULTS** |  |  |  |
| **Study selection** | 16a | Describe the results of the search and selection process, from the number of records identified in the search to the number of studies included in the review, ideally using a flow diagram. | The results of the search process are detailed, including the number of records identified (943), screened, and the final 26 studies included in the meta-analysis. This is represented using a PRISMA flow diagram​ |
|  | 16b | Cite studies that might appear to meet the inclusion criteria, but which were excluded, and explain why they were excluded. | Reasons for exclusion of studies are provided, such as wrong outcome, wrong study design, no control group, etc. These are also listed in the supplementary materials​ |
| **Study characteristics** | 17 | Cite each included study and present its characteristics. | The characteristics of the included studies, such as population size, intervention details, and follow-up times, are presented in a table format​ |
| **Risk of bias in studies** | 18 | Present assessments of risk of bias for each included study. | Risk of bias assessments for each included study are presented using the ROBINS-I tool, with results visually represented in figures​ |
| **Results of individual studies** | 19 | For all outcomes, present, for each study: (a) summary statistics for each group (where appropriate) and (b) an effect estimate and its precision (e.g. confidence/credible interval), ideally using structured tables or plots. | The results for individual studies are presented with summary statistics, risk ratios, and confidence intervals. These are shown in forest plots for each outcome |
| **Results of syntheses** | 20a | For each synthesis, briefly summarise the characteristics and risk of bias among contributing studies. | The meta-analysis synthesizes the outcomes, highlighting mortality and adverse effects, with a summary of study characteristics and risk of bias |
|  | 20b | Present results of all statistical syntheses conducted. If meta-analysis was done, present for each the summary estimate and its precision (e.g. confidence/credible interval) and measures of statistical heterogeneity. If comparing groups, describe the direction of the effect. | The summary estimates with confidence intervals for mortality and adverse effects are presented in the results section using forest plots​ |
|  | 20c | Present results of all investigations of possible causes of heterogeneity among study results. | Heterogeneity was assessed using I² statistics, and results are presented for each outcome​ |
|  | 20d | Present results of all sensitivity analyses conducted to assess the robustness of the synthesized results. | Sensitivity analyses were conducted and presented to assess the robustness of the synthesized results by removing outlier studies​ |
| **Reporting biases** | 21 | Present assessments of risk of bias due to missing results (arising from reporting biases) for each synthesis assessed. | Reporting bias was assessed using funnel plots and Egger’s test, and the results are presented in the supplementary material​ |
| **Certainty of evidence** | 22 | Present assessments of certainty (or confidence) in the body of evidence for each outcome assessed. | Certainty was discussed in relation to the sensitivity analyses and the strength of the evidence for different outcomes |
| **DISCUSSION** |  |  |  |
| **Discussion** | 23a | Provide a general interpretation of the results in the context of other evidence. | The discussion provides an interpretation of the results, highlighting that LV unloading strategies (especially IABP) are associated with reduced mortality in patients undergoing VA-ECMO and comparing these findings with previous studies |
|  | 23b | Discuss any limitations of the evidence included in the review. | The limitations include the retrospective nature of most included studies, limited control over confounding factors, and lack of randomised controlled trials​ |
|  | 23c | Discuss any limitations of the review processes used. | The discussion acknowledges that most studies were observational, and the review relied on available retrospective data, which may have introduced bias |
|  | 23d | Discuss implications of the results for practice, policy, and future research. | The conclusion discusses the clinical implications of using LV unloading strategies, especially IABP, and highlights the need for further randomised controlled trials to establish optimal management protocols |
| **OTHER INFORMATION** |  |  |  |
| **Registration and protocol** | 24a | Provide registration information for the review, including register name and registration number, or state that the review was not registered. | The review is registered on PROSPERO with the registration number CRD42024580078 |
|  | 24b | Indicate where the review protocol can be accessed, or state that a protocol was not prepared. | The review protocol is not explicitly mentioned as being available separately for access​ |
|  | 24c | Describe and explain any amendments to information provided at registration or in the protocol. | Describe and explain any amendments to information provided at registration or in the protocol. |
| **Support** | 25 | Describe sources of financial or non-financial support for the review, and the role of the funders or sponsors in the review. | N/A |
| **Competing interests** | 26 | Declare any competing interests of review authors. | N/A |
| **Availability of data, code and other materials** | 27 | Report which of the following are publicly available and where they can be found: template data collection forms; data extracted from included studies; data used for all analyses; analytic code; any other materials used in the review. | Supplementary Materials |

#####

# PRIMSA Abstract Checklist

| **Topic** | **No.** | **Item** | **Reported?** |
| --- | --- | --- | --- |
| **TITLE** |  |  |  |
| **Title** | 1 | Identify the report as a systematic review. | Yes |
| **BACKGROUND** |  |  |  |
| **Objectives** | 2 | Provide an explicit statement of the main objective(s) or question(s) the review addresses. | Yes |
| **METHODS** |  |  |  |
| **Eligibility criteria** | 3 | Specify the inclusion and exclusion criteria for the review. | Yes |
| **Information sources** | 4 | Specify the information sources (e.g. databases, registers) used to identify studies and the date when each was last searched. | Yes |
| **Risk of bias** | 5 | Specify the methods used to assess risk of bias in the included studies. | Yes |
| **Synthesis of results** | 6 | Specify the methods used to present and synthesize results. | Yes |
| **RESULTS** |  |  |  |
| **Included studies** | 7 | Give the total number of included studies and participants and summarise relevant characteristics of studies. | Yes |
| **Synthesis of results** | 8 | Present results for main outcomes, preferably indicating the number of included studies and participants for each. If meta-analysis was done, report the summary estimate and confidence/credible interval. If comparing groups, indicate the direction of the effect (i.e. which group is favoured). | Yes |
| **DISCUSSION** |  |  |  |
| **Limitations of evidence** | 9 | Provide a brief summary of the limitations of the evidence included in the review (e.g. study risk of bias, inconsistency and imprecision). | Yes |
| **Interpretation** | 10 | Provide a general interpretation of the results and important implications. | Yes |
| **OTHER** |  |  |  |
| **Funding** | 11 | Specify the primary source of funding for the review. | Yes |
| **Registration** | 12 | Provide the register name and registration number. | Yes |

**Annex A; Search Strategy**

1. impella.mp. [mp=ti, ab, hw, tn, ot, dm, mf, dv, kf, fx, dq, bt, nm, ox, px, rx, an, ui, sy, ux, mx]
2. iabp.mp. [mp=ti, ab, hw, tn, ot, dm, mf, dv, kf, fx, dq, bt, nm, ox, px, rx, an, ui, sy, ux, mx]
3. VA-ECMO.mp. [mp=ti, ab, hw, tn, ot, dm, mf, dv, kf, fx, dq, bt, nm, ox, px, rx, an, ui, sy, ux, mx]
4. transaortic catheter.mp. [mp=ti, ab, hw, tn, ot, dm, mf, dv, kf, fx, dq, bt, nm, ox, px, rx, an, ui, sy, ux, mx]
5. 1 or 2 or 4
6. 3 and 5
7. remove duplicates from 6
8. limit 7 to English language
9. limit 8 to human
10. limit 9 to humans

**Table S.1: Studies Excluded in Full Text Screening**

| **Author** | **Study Name** | **DOI** | **Reason for Exclusion** |
| --- | --- | --- | --- |
| Modi S.P. | Concomitant Use of VA-ECMO and Impella Support for Cardiogenic Shock | https://dx.doi.org/10.1101/2023.07.24.23293127 | No Control |
| Ullah K | Complex and High-Risk Percutaneous Intervention Assisted By Extracorporeal Membrane Oxygenation (ECMO) | https://dx.doi.org/10.1101/2023.05.26.23290621 | Wrong Outcome |
| Kang J. | Differential effect of left ventricular unloading according to the aetiology of cardiogenic shock | https://dx.doi.org/10.1002/ehf2.14584 | Wrong Outcome |
| Alnasser S. | Utilization of Shock Team and Veno-Arterial Extracorporeal Membrane Oxygenation (VA-ECMO) in the Management of Cardiogenic Shock in Northern Ontario | https://dx.doi.org/10.1016/j.cjco.2023.09.019 | Wrong Design |
| Sakiyalak P. | Long-term survival outcome and the quality of life of patients with postcardiotomy venoarterial extracorporeal membrane oxygenator | - | Wrong Design |
| Bogerd M. | Impella and venoarterial extracorporeal membrane oxygenation in cardiogenic shock complicating acute myocardial infarction | https://dx.doi.org/10.1002/ejhf.3025 | Wrong Design |
| Ahmad S | Effect of Mechanical Unloading on Cardiogenic Shock Severity in Venoarterial Extracorporeal Membrane Oxygenation Patients | https://dx.doi.org/10.1161/circ.148.suppl_1.18693 | Conference Abstract |
| Yeo I. | Comparative Outcomes of Impella and Intra-Aortic Balloon Pump for Left Ventricular Unloading Among Patients With Cardiogenic Shock on Veno-Arterial Extracorporeal Membrane Oxygenation | <https://dx.doi.org/10.1161/circ.148.suppl_1.11322> | Conference Abstract |
| Kieserman J. | Comparing Veno-arterial Extracorpeal Membrane Oxygenation (VAECMO) vs. VA-ECMO plus Impella (ECPELLA) on Mortality in Patients with Cardiac Arrest After an Acute Myocardial Infarction (AMI) | https://dx.doi.org/10.1097/01.mat.0000943300.58975.7e | Conference Abstract |
| Thevathasan T. | Left-ventricular unloading in extracorporeal cardiopulmonary resuscitation due to acute myocardial infarction - A multicenter study | https://dx.doi.org/10.1016/j.resuscitation.2023.109775 | Duplicate |
| Thevathasan T. | Treatment with Impella and veno-arterial extracorporeal membrane oxygenation during cardiac arrest on survival in a multicenter cohort | https://dx.doi.org/10.1093/eurheartj/ehac544.1472 | Conference Abstract |
| Denisenko A. | Left ventricular unloading did not decrease mortality in cardiogenic shocks patients supported with VA-ECMO: A propensity score matching study | https://dx.doi.org/10.1016/j.accpm.2022.101042 | Wrong Outcome |
| Udongwo N. | Does Concomitant Left Ventricular Unloading Improve Outcomes Of Cardiogenic Shock Managed By Venoarterial Extracorporeal Membrane Oxygenation? Analysis Of National Inpatient Sample Database 2019 | https://dx.doi.org/10.1161/circ.146.suppl_1.332 | Conference Abstract |
| Thevathasan T. | Treatment with impella and veno-arterial extracorporeal membrane oxygenation during extracorporeal cardiopulmonary resuscitation improves survival-a multicenter cohort study | https://dx.doi.org/10.1161/circ.144.suppl-1.12405 | Duplicate |
| Kurpad K.K. | Outcomes of extracorporeal membrane oxygenation with or without left ventricular unloading devices in patients with non-myocardial infarction cardiogenic shock | https://dx.doi.org/10.1093/eurheartj/ehab724.1057 | Conference Abstract |
| Manla Y. | Utilization of left ventricular unloading in VA-ECMO patients and its association with intensive care length of stay and survival | https://dx.doi.org/10.1002/ejhf.2297 | Conference Abstract |
| Christopher Gaisendrees C. | Impact of left ventricular unloading using peripheral Impella in eCPR patients | https://dx.doi.org/10.1002/ejhf.2297 | Duplicate |
| Young B.A.C. | Risk Factors and Outcomes of Patients Requiring Left Ventricular Unloading on Veno-Arterial Extracorporeal Membrane Oxygenation Support | https://dx.doi.org/10.1097/MAT.0000000000001492 | Conference Abstract |
| Kurpad K.P. | Va-ecmo vs va ecmo plus iabp in non-mi cardiogenic shock: Outcomes analysing the nationwide inpatient sample 2015-2018 | https://dx.doi.org/10.1002/ccd.29644 | Conference Abstract |
| Char S. | Clinical and Hemodynamic Efficacy of Left Ventricular Unloading during Venoarterial Extracorporeal Membrane Oxygenation | https://dx.doi.org/10.1016/j.healun.2021.01.1139 | Duplicate |
| Schrage B. | Left Ventricular Unloading Is Associated With Lower Mortality in Patients With Cardiogenic Shock Treated With Venoarterial Extracorporeal Membrane Oxygenation Results From an International, Multicenter Cohort Study | https://dx.doi.org/10.1161/CIRCULATIONAHA.120.048792 | Duplicate |
| Tamura Y. | Combined Use of VA-ECMOecmo and Impella (ECPELLA) Improves Outcome of Patients with out of Hospital Cardiac Arrest or Cardiac Arrest at the Emergency Room | https://dx.doi.org/10.1161/circ.142.suppl_4.268 | Conference Abstract |
| Ansari S. | Cardiogenic shock patients on va-ECMO with and without unloading from impella | https://dx.doi.org/10.1177/1556984520951281d | Conference Abstract |
| Unoki T. | Combined use of VA-ECMO and IMPELLA (ECPELLA) as a possible strategy to improve outcomes in patients who underwent E-CPR | https://dx.doi.org/10.1093/ehjci/ehaa946.1861 | Duplicate |
| Kang J. | Differential effect of left ventricular unloading according to the aetiology of cardiogenic shock | https://dx.doi.org/10.1002/ehf2.14584 | Wrong Design |
| Amancherla K. | A Single-Center Experience with Balloon Atrial Septostomy as an Unloading Strategy in Patients on ECMO | https://dx.doi.org/10.1016/j.healun.2020.01.197 | Conference Abstract |
| Piechura L. | Retrospective review of left ventricular unloading strategies during veno-arterial extracorporeal membrane oxygenation | https://dx.doi.org/10.1097/MAT.0000000000001007 | Conference Abstract |
| Zaid S. | TCT-815 Does Addition of Percutaneous Left Ventricular Decompression Therapy With Impella Affect Clinical Outcomes in Cardiogenic Shock Patients on Venoarterial Extracorporeal Membrane Oxygenation? A Nationwide Analysis | https://dx.doi.org/10.1016/j.jacc.2019.08.960 | Conference Abstract |
| Delmas C. | Place of left heart decompression in patients under veno-arterial ECMO: Benefits of percutaneous balloon atrioseptostomy? | <https://dx.doi.org/10.1177/2048872619829424> | Conference Abstract |
| Fuerholz M. | Temporary mechanical circulatory support systems in current clinical practice: Indication and clinical outcome in a tertiary care center | https://dx.doi.org/10.1177/2048872619829424 | Conference Abstract |
| Singh A. | Impact of left ventricular decompression with impella during venoarterial extracorporeal membrane oxygenation support in acute myocardial infarction complicated by cardiogenic shock | https://dx.doi.org/10.1002/ccd.28216 | Conference Abstract |
| Fiedler A.G. | Impella Placement Guided by Echocardiography Can Be Used as a Strategy to Unload the Left Ventricle During Peripheral Venoarterial Extracorporeal Membrane Oxygenation | https://dx.doi.org/10.1053/j.jvca.2018.05.019 | Wrong Design |
| Eliet J. | Effect of impella during veno-arterial extracorporeal membrane oxygenation on pulmonary artery flow as assessed by end-tidal carbon dioxide | https://dx.doi.org/10.1097/MAT.0000000000000662 | Wrong Outcome |
| Morine K.J. | Clinical outcomes among patients requiring acute mechanical circulatory support for cardiogenic shock supported by impella or VA-ECMO | https://dx.doi.org/10.1097/MAT.0000000000000814 | Wrong Design |
| Morine K.J. | Congestive profiles correlate with clinical outcomes among patients requiring acute mechanical circulatory support for cardiogenic shock | https://dx.doi.org/10.1097/MAT.0000000000000814 | Conference Abstract |
| Morine K. | Congestive profiles correlate with clinical outcomes among patients requiring acute mechanical circulatory support for cardiogenic shock | https://dx.doi.org/10.1016/j.jcin.2018.01.021 | Duplicate |
| Morine K. | Clinical outcomes among patients requiring acute mechanical circulatory support for cardiogenic shock supported by impella or VA-ECMO | https://dx.doi.org/10.1016/j.jcin.2018.01.018 | Duplicate |
| Kuchibhotla S. | Utility of the SAVE score as a predictor of survival in cardiogenic shock requiring VA-ECMO or impella for acute mechanical circulatory support | - | Wrong Design |
| Tongers J. | Dual mechanical support combining impella microaxial pump and veno-arterial ECMO rescues high-risk patients in refractory cardiogenic shock | - | Wrong Design |
| Esposito M. | Comparing hemodynamic profiles and outcomes in cardiogenic shock requiring VA-ECMO or Impella for circulatory support | - | Wrong Design |
| Negi S.I | A comparative analysis of use of extracorporeal membrane oxygenation and peripheral ventricular assist device tandemheart in acute myocardial infarction | - | Wrong Design |
| Cheng A. | Impella to unload the left ventricle during peripheral extracorporeal membrane oxygenation | https://dx.doi.org/10.1097/MAT.0b013e31829f0e52 | Wrong Design |
| Wang J.-G. | Outcome of Veno-Arterial Extracorporeal Membrane Oxygenation for Patients Undergoing Valvular Surgery | https://dx.doi.org/10.1371/journal.pone.0063924 | Wrong Design |
| Nitta M. | In-Hospital Mortality in Patients With Cardiogenic Shock Requiring Veno-Arterial Extracorporeal Membrane Oxygenation With Concomitant Use of Impella vs. Intra-Aortic Balloon Pump - A Retrospective Cohort Study Using a Japanese Claims-Based Database | https://dx.doi.org/10.1253/circj.CJ-23-0758 | No Control |
| Inglis S.S. | Novel Left Ventricular Unloading Strategies in Patients on Peripheral Venoarterial Extracorporeal Membrane Oxygenation Support | https://dx.doi.org/10.1097/MAT.0000000000002136 | No Control |

References:

Ahmad, S, et al. “Abstract 18693: Effect of Mechanical Unloading on Cardiogenic Shock Severity in Venoarterial Extracorporeal Membrane Oxygenation Patients.” Circulation, vol. 148, no. Suppl_1, 7 Nov. 2023, https://doi.org/10.1161/circ.148.suppl_1.18693. Accessed 10 Sept. 2024.

Ansari, S. “Abstracts.” Innovations: Technology and Techniques in Cardiothoracic and Vascular Surgery, vol. 15, no. 1_suppl, Oct. 2020, pp. 6S60S, https://doi.org/10.1177/1556984520951281d. Accessed 3 Feb. 2023.

Char, S, et al. “Clinical and Hemodynamic Efficacy of Left Ventricular Unloading during Venoarterial Extracorporeal Membrane Oxygenation.” The Journal of Heart and Lung Transplantation, vol. 40, no. 4, 1 Apr. 2021, pp. S406–S406, https://doi.org/10.1016/j.healun.2021.01.1139. Accessed 10 Sept. 2024.

Cheng, Allen, et al. “Impella to Unload the Left Ventricle during Peripheral Extracorporeal Membrane Oxygenation.” ASAIO Journal, vol. 59, no. 5, 2013, pp. 533–536, https://doi.org/10.1097/mat.0b013e31829f0e52. Accessed 29 Oct. 2020.

Delmas, C. “Acute Cardiovascular Care 2019.” European Heart Journal: Acute Cardiovascular Care, vol. 8, no. 1_suppl, Apr. 2019, pp. 5–440, https://doi.org/10.1177/2048872619829424. Accessed 23 Apr. 2020.

Denisenko, Alina, et al. “Left Ventricular Unloading Did Not Decrease Mortality in Cardiogenic Shocks Patients Supported with VA-ECMO: A Propensity Score Matching Study.” Anaesthesia Critical Care & Pain Medicine, vol. 41, no. 2, 1 Apr. 2022, pp. 101042–101042, https://doi.org/10.1016/j.accpm.2022.101042. Accessed 10 Sept. 2024.

Eliet, Jacob, et al. “Effect of Impella during Veno-Arterial Extracorporeal Membrane Oxygenation on Pulmonary Artery Flow as Assessed by End-Tidal Carbon Dioxide.” ASAIO Journal, vol. 64, no. 4, July 2018, pp. 502–507, https://doi.org/10.1097/mat.0000000000000662. Accessed 14 Nov. 2022.

Fiedler, Amy G, et al. “Impella Placement Guided by Echocardiography Can Be Used as a Strategy to Unload the Left Ventricle during Peripheral Venoarterial Extracorporeal Membrane Oxygenation.” Journal of Cardiothoracic and Vascular Anesthesia, vol. 32, no. 6, 1 Dec. 2018, pp. 2585–2591, https://doi.org/10.1053/j.jvca.2018.05.019. Accessed 8 Nov. 2023.

Fuerholz, M. “Acute Cardiovascular Care 2019.” European Heart Journal: Acute Cardiovascular Care, vol. 8, no. 1_suppl, Apr. 2019, pp. 5–440, https://doi.org/10.1177/2048872619829424. Accessed 23 Apr. 2020.

Gaisendrees, Christopher, et al. “Impact of Left Ventricular Unloading Using a Peripheral Impella®‐Pump in ECPR Patients.” Artificial Organs, vol. 46, no. 3, 25 Sept. 2021, pp. 451–459, https://doi.org/10.1111/aor.14067.

Inglis, Sara S, et al. “Novel Left Ventricular Unloading Strategies in Patients on Peripheral Venoarterial Extracorporeal Membrane Oxygenation Support.” ASAIO Journal, vol. 70, no. 5, 2 Jan. 2024, pp. 396–403, https://doi.org/10.1097/mat.0000000000002136. Accessed 10 Sept. 2024.

K. Amancherla, et al. “A Single-Center Experience with Balloon Atrial Septostomy as an Unloading Strategy in Patients on ECMO.” The Journal of Heart and Lung Transplantation, vol. 39, no. 4, 1 Apr. 2020, pp. S420–S420, https://doi.org/10.1016/j.healun.2020.01.197. Accessed 10 Sept. 2024.

Kang, Jeehoon, et al. “Differential Effect of Left Ventricular Unloading according to the Aetiology of Cardiogenic Shock.” ESC Heart Failure, vol. 11, no. 1, 27 Nov. 2023, pp. 338–348, https://doi.org/10.1002/ehf2.14584. Accessed 8 Aug. 2024.

Kieserman, Jake, et al. “SHOCK3: Comparing Veno-Arterial Extracorpeal Membrane Oxygenation (VA-ECMO) vs. VA-ECMO plus Impella (ECPELLA) on Mortality in Patients with Cardiac Arrest after an Acute Myocardial Infarction (AMI).” ASAIO Journal, vol. 69, no. Supplement 2, 1 June 2023, pp. 10–10, https://doi.org/10.1097/01.mat.0000943300.58975.7e. Accessed 10 Sept. 2024.

Kurpad, K K, et al. “Outcomes of Extracorporeal Membrane Oxygenation with or without Left Ventricular Unloading Devices in Patients with Non-Myocardial Infarction Cardiogenic Shock.” European Heart Journal, vol. 42, no. Supplement_1, 1 Oct. 2021, https://doi.org/10.1093/eurheartj/ehab724.1057. Accessed 10 Sept. 2024.

Kurpad, K.P. “Abstract.” Catheterization and Cardiovascular Interventions, vol. 97, no. S1, 15 Apr. 2021, https://doi.org/10.1002/ccd.29644.

Manla, Yosef. “Utilization of Left Ventricular Unloading in VA-ECMO Patients and Its Association with Intensive Care Length of Stay and Survival.” Escardio.org, 2021, esc365.escardio.org/presentation/234000. Accessed 10 Sept. 2024.

Margriet Bogerd, et al. “Impella and Venoarterial Extracorporeal Membrane Oxygenation in Cardiogenic Shock Complicating Acute Myocardial Infarction.” European Journal of Heart Failure, vol. 25, no. 11, 18 Sept. 2023, pp. 2021–2031, https://doi.org/10.1002/ejhf.3025. Accessed 12 May 2024.

Modi SP, Hong Y, Sicke MM, Hess NR, Klass WJ, Ziegler LA, et al. Concomitant Use of VA-ECMO and Impella Support for Cardiogenic Shock. medRxiv (Cold Spring Harbor Laboratory). 2023 Jul 27;

Modi, Shan P, et al. “Concomitant Use of VA-ECMO and Impella Support for Cardiogenic Shock.” MedRxiv (Cold Spring Harbor Laboratory), 27 July 2023, https://doi.org/10.1101/2023.07.24.23293127.

Morine, K.J. “ASAIO 2018 Annual Meeting Abstracts.” ASAIO Journal, vol. 64, 2018, pp. 1–143, https://doi.org/10.1097/mat.0000000000000814. Accessed 18 May 2020.

Morine, K.J. “ASAIO 2018 Annual Meeting Abstracts.” ASAIO Journal, vol. 64, 2018, pp. 1–143, https://doi.org/10.1097/mat.0000000000000814. Accessed 18 May 2020.

Morine, Kevin, et al. “CRT-100.07 Clinical Outcomes among Patients Requiring Acute Mechanical Circulatory Support for Cardiogenic Shock Supported by Impella or VA-ECMO.” JACC: Cardiovascular Interventions, vol. 11, no. 4, Feb. 2018, p. S3, https://doi.org/10.1016/j.jcin.2018.01.018. Accessed 31 Aug. 2021.

Morine, Kevin, et al. “CRT-100.10 Congestive Profiles Correlate with Clinical Outcomes among Patients Requiring Acute Mechanical Circulatory Support for Cardiogenic Shock.” JACC: Cardiovascular Interventions, vol. 11, no. 4, Feb. 2018, p. S4, https://doi.org/10.1016/j.jcin.2018.01.021. Accessed 31 Aug. 2021.

Ndausung Udongwo, et al. “Abstract 332: Does Concomitant Left Ventricular Unloading Improve Outcomes of Cardiogenic Shock Managed by Venoarterial Extracorporeal Membrane Oxygenation? Analysis of National Inpatient Sample Database 2019.” Circulation, vol. 146, no. Suppl_1, 8 Nov. 2022, https://doi.org/10.1161/circ.146.suppl_1.332. Accessed 10 Sept. 2024.

Nitta, Manabu, et al. “In-Hospital Mortality in Patients with Cardiogenic Shock Requiring Veno-Arterial Extracorporeal Membrane Oxygenation with Concomitant Use of Impella vs. Intra-Aortic Balloon Pump ― a Retrospective Cohort Study Using a Japanese Claims-Based Database ―.” Circulation Journal, vol. 88, no. 8, 25 July 2024, pp. 1276–1285, https://doi.org/10.1253/circj.cj-23-0758. Accessed 10 Sept. 2024.

Piechura, L. “ASAIO 2019 Annual Meeting Abstracts.” ASAIO Journal, vol. 65, no. Supplement 1, May 2019, pp. 1–147, https://doi.org/10.1097/mat.0000000000001007.

Sami Alnasser, et al. “Utilization of Shock Team and Veno-Arterial Extracorporeal Membrane Oxygenation (VA-ECMO) in the Management Cardiogenic Shock in North Ontario.” CJC Open, vol. 6, no. 1, 1 Jan. 2024, pp. 47–53, https://doi.org/10.1016/j.cjco.2023.09.019. Accessed 10 Sept. 2024.

Schrage, Benedikt, et al. “Left Ventricular Unloading Is Associated with Lower Mortality in Patients with Cardiogenic Shock Treated with Venoarterial Extracorporeal Membrane Oxygenation.” Circulation, vol. 142, no. 22, Dec. 2020, pp. 2095–2106, https://doi.org/10.1161/circulationaha.120.048792. Accessed 12 Dec. 2020.

Singh, A. “Abstracts.” Catheterization and Cardiovascular Interventions, vol. 93, no. S2, May 2019, pp. S1–S246, https://doi.org/10.1002/ccd.28216. Accessed 25 June 2022.

T Thevathasan, et al. “Treatment with Impella and Veno-Arterial Extracorporeal Membrane Oxygenation during Cardiac Arrest on Survival in a Multicenter Cohort.” European Heart Journal, vol. 43, no. Supplement_2, 1 Oct. 2022, https://doi.org/10.1093/eurheartj/ehac544.1472. Accessed 10 Sept. 2024.

T Unoki, et al. “Combined Use of VA-ECMO and IMPELLA (ECPELLA) as a Possible Strategy to Improve Outcomes in Patients Who Underwent E-CPR.” European Heart Journal, vol. 41, no. Supplement_2, 1 Nov. 2020, https://doi.org/10.1093/ehjci/ehaa946.1861. Accessed 10 Sept. 2024.

Tamura, Yudai, et al. “Abstract 268: Combined Use of VA-ECMOecmo and Impella (ECPELLA) Improves Outcome of Patients with out of Hospital Cardiac Arrest or Cardiac Arrest at the Emergency Room.” Circulation, vol. 142, no. Suppl_4, 17 Nov. 2020, https://doi.org/10.1161/circ.142.suppl_4.268. Accessed 10 Sept. 2024.

Tharusan Thevathasan, et al. Left-Ventricular Unloading in Extracorporeal Cardiopulmonary Resuscitation due to Acute Myocardial Infarction – a Multicenter Study. Vol. 186, 1 May 2023, pp. 109775–109775, https://doi.org/10.1016/j.resuscitation.2023.109775. Accessed 26 June 2023.

Tharusan Thevathasan, et al. “Abstract 12405: Treatment with Impella and Veno-Arterial Extracorporeal Membrane Oxygenation during Extracorporeal Cardiopulmonary Resuscitation Improves Survival - a Multicenter Cohort Study.” Circulation, vol. 144, no. Suppl_1, 16 Nov. 2021, https://doi.org/10.1161/circ.144.suppl_1.12405. Accessed 10 Sept. 2024.

Ullah, Kifayat, and Liu Bin. “Complex and High-Risk Percutaneous Intervention Assisted by Extracorporeal Membrane Oxygenation (ECMO).” MedRxiv (Cold Spring Harbor Laboratory), 4 June 2023, https://doi.org/10.1101/2023.05.26.23290621. Accessed 10 Sept. 2024.

Wang, Jian-Gang, et al. “Outcome of Veno-Arterial Extracorporeal Membrane Oxygenation for Patients Undergoing Valvular Surgery.” PLoS ONE, vol. 8, no. 5, 23 May 2013, p. e63924, https://doi.org/10.1371/journal.pone.0063924. Accessed 13 Mar. 2022.

Yeo, Ilhwan, et al. “Abstract 11322: Comparative Outcomes of Impella and Intra-Aortic Balloon Pump for Left Ventricular Unloading among Patients with Cardiogenic Shock on Veno-Arterial Extracorporeal Membrane Oxygenation.” Circulation, vol. 148, no. Suppl_1, 7 Nov. 2023, https://doi.org/10.1161/circ.148.suppl_1.11322. Accessed 10 Sept. 2024.

Young, B.A.C. “ASAIO 2021 Annual Meeting Abstracts.” ASAIO Journal, vol. 67, no. Supplement 2, June 2021, pp. 1–155, https://doi.org/10.1097/mat.0000000000001492. Accessed 24 July 2022.

Zaid, Syed, et al. “TCT-815 Does Addition of Percutaneous Left Ventricular Decompression Therapy with Impella Affect Clinical Outcomes in Cardiogenic Shock Patients on Venoarterial Extracorporeal Membrane Oxygenation? A Nationwide Analysis.” Journal of the American College of Cardiology, vol. 74, no. 13, 1 Oct. 2019, pp. B798–B798, https://doi.org/10.1016/j.jacc.2019.08.960.

**Figure S.2:** Funnel Plot Overview of Studies – outcome all-cause mortality


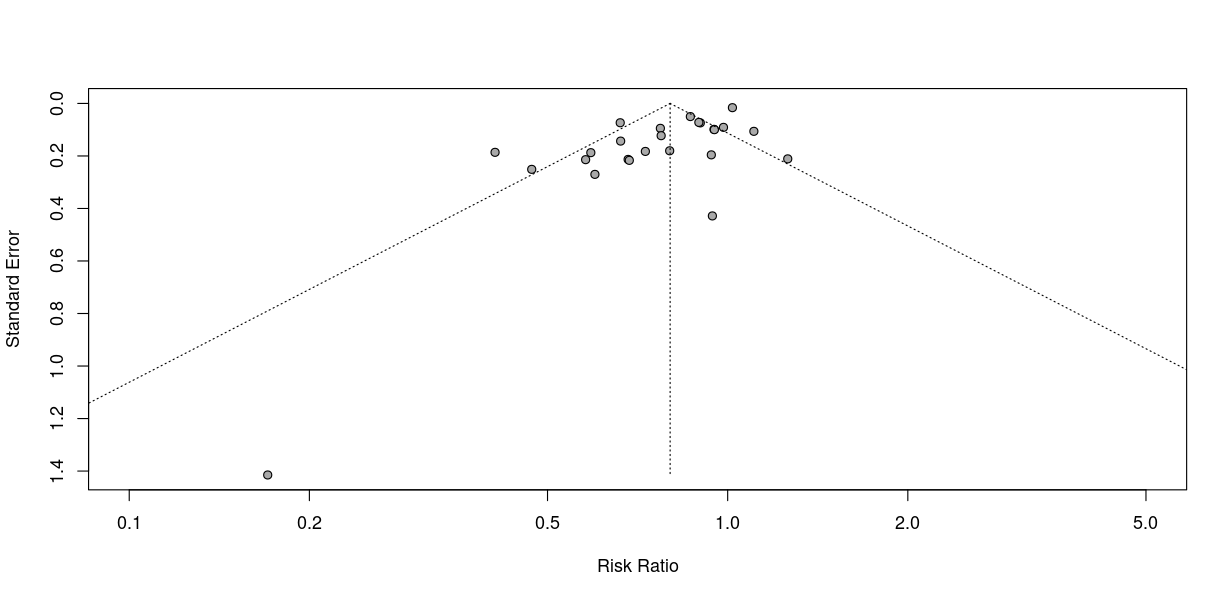


**Figure S.3:** Forest-plots for the outcome Intra-Hospital Mortality: Panel A - Impella compared to no unloading; Panel B: IABP compared to no unloading; and Panel C: unloading compared no unloading

**
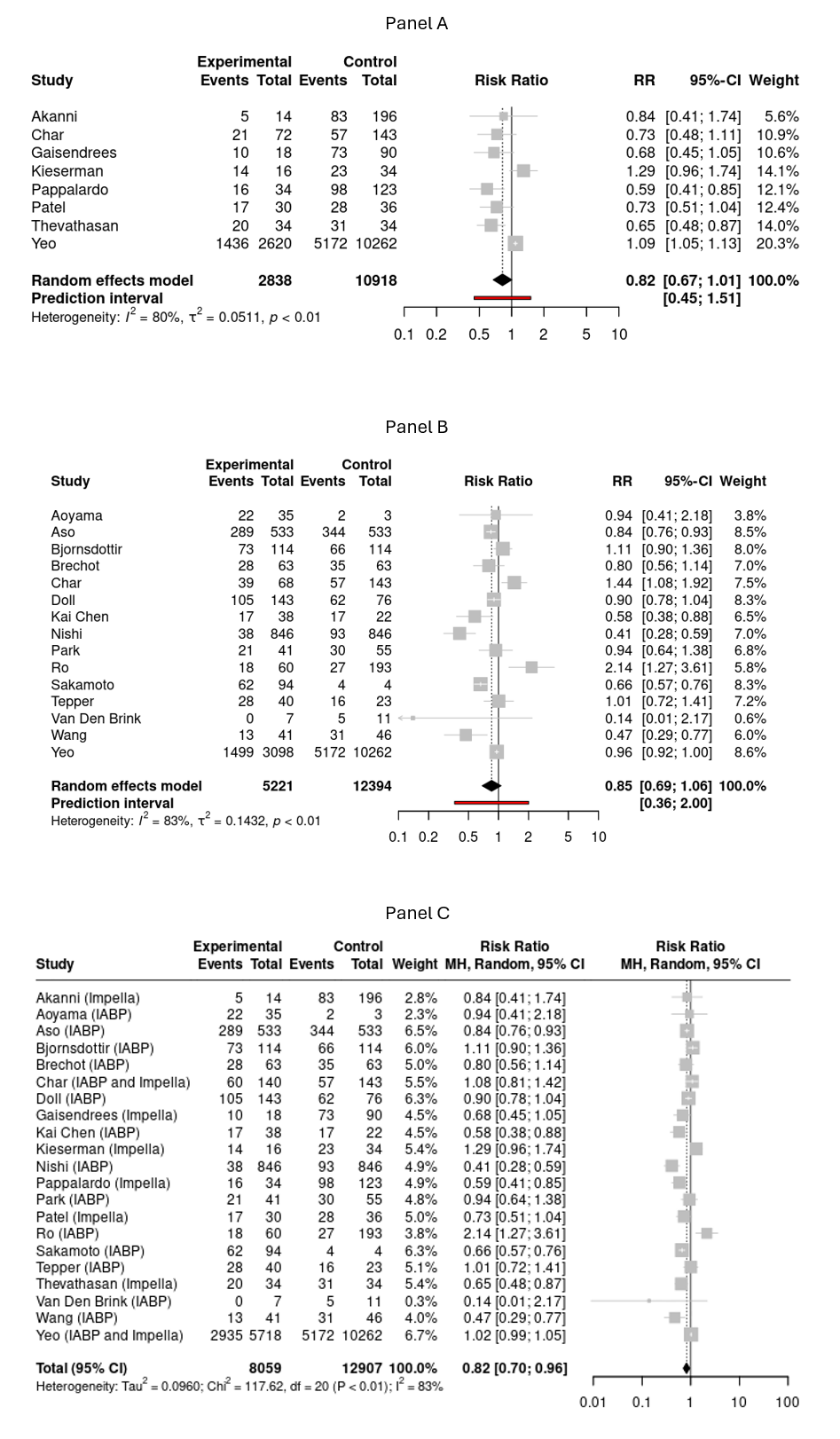
**

**Figure S.4:** Forest-plots for the outcome 30 Day Mortality: Panel A - Impella compared to no unloading; Panel B: IABP compared to no unloading; and Panel C: unloading compared no unloading

**
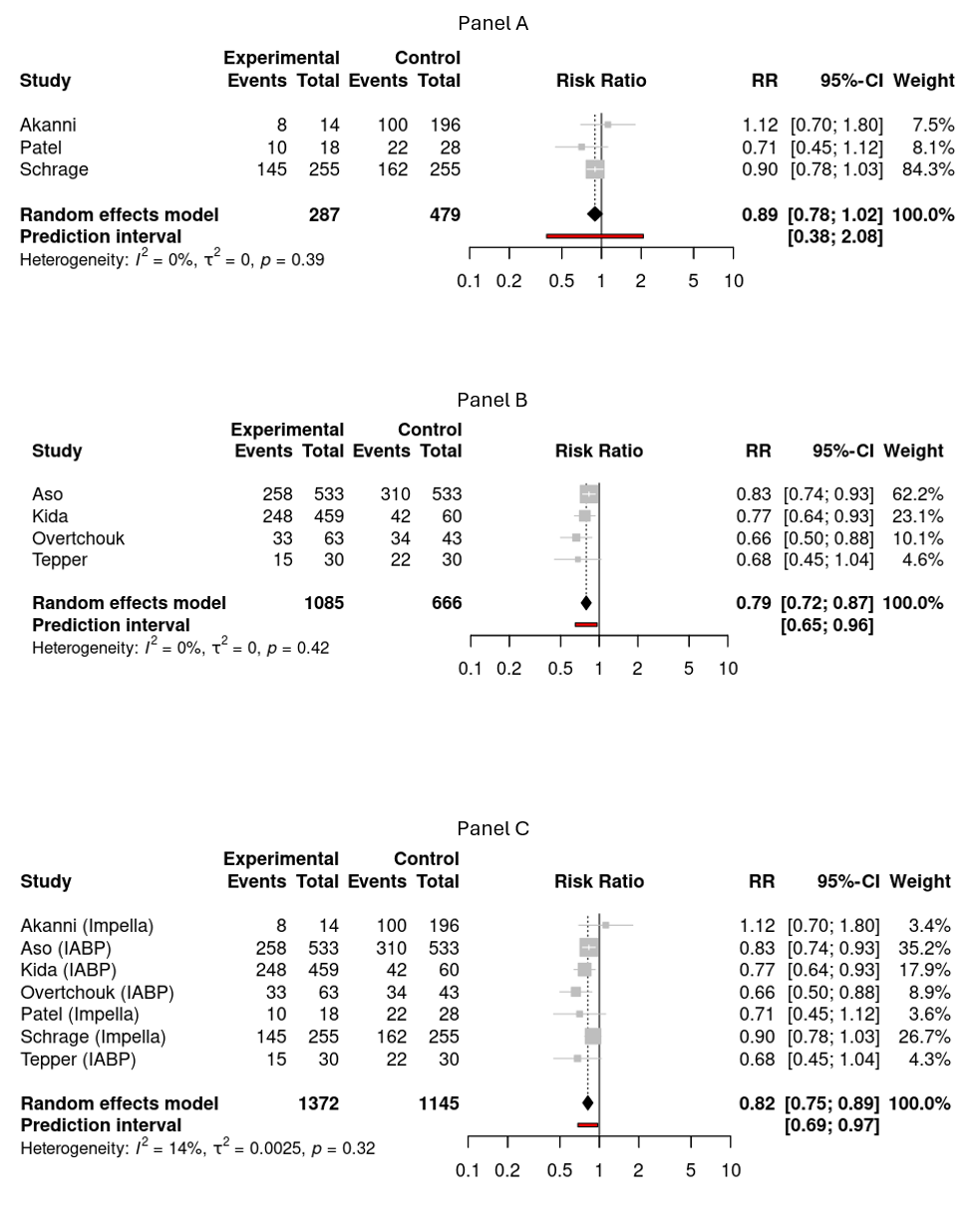
**

**Figure S.5:** Forest-plots for the outcome bleeding: Panel A - Impella compared to no unloading; Panel B: IABP compared to no unloading; Panel C: IABP compared to Impella; and Panel D: unloading compared no unloading


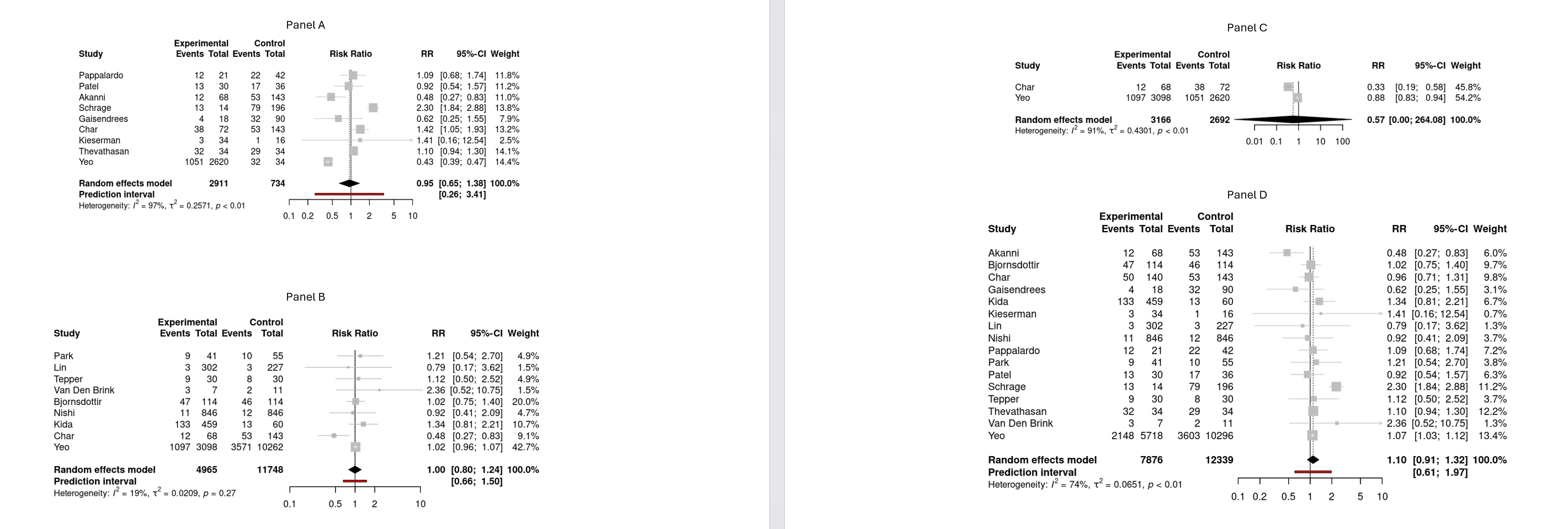


**Figure S.6:** Forest-plots for the outcome CVA: Panel A - Impella compared to no unloading; Panel B: IABP compared to no unloading; Panel C: IABP compared to Impella; and Panel D: unloading compared no unloading


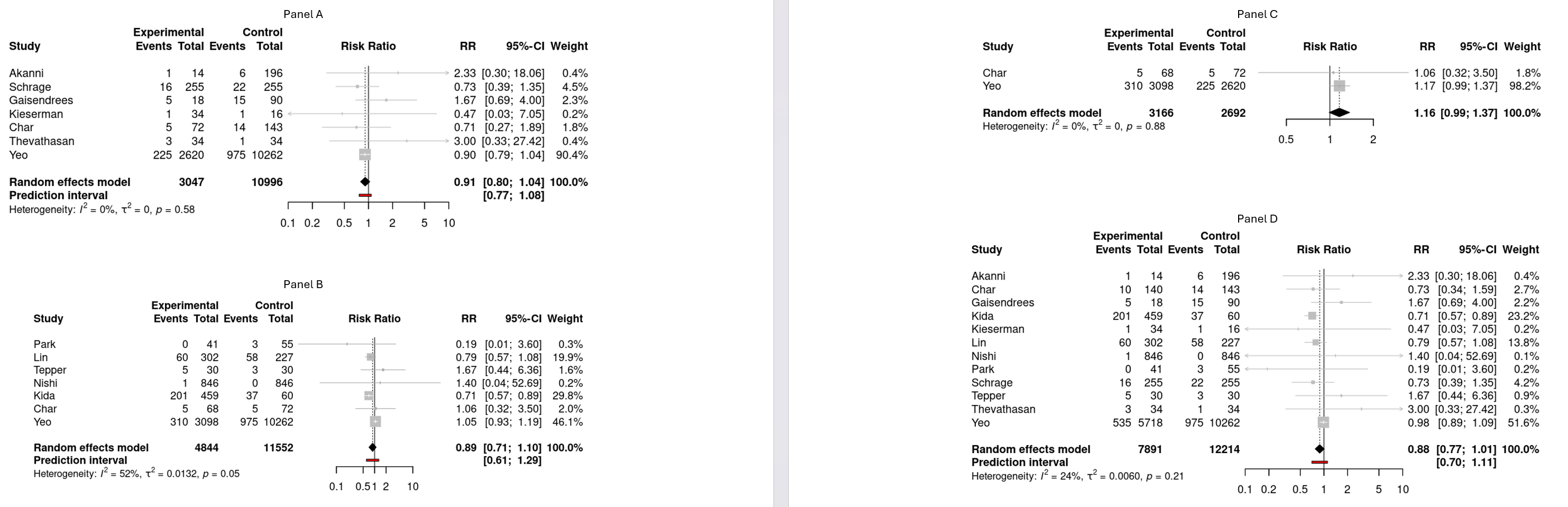


**Figure S.7:** Forest-plots for the outcome limb ischaemia: Panel A - Impella compared to no unloading; Panel B: IABP compared to no unloading; Panel C: IABP compared to Impella; and Panel D: unloading compared no unloading


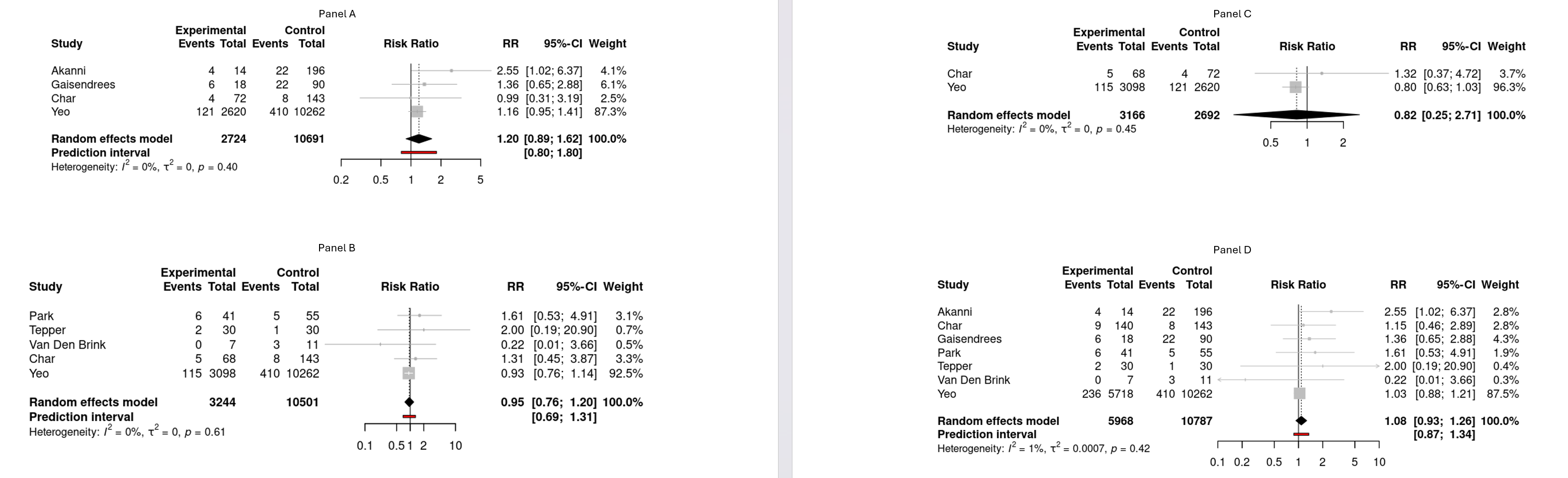


**Figure S.8:** Forest-plots for the outcome RRT: Panel A - Impella compared to no unloading; Panel B: IABP compared to no unloading; and Panel C: unloading compared no unloading


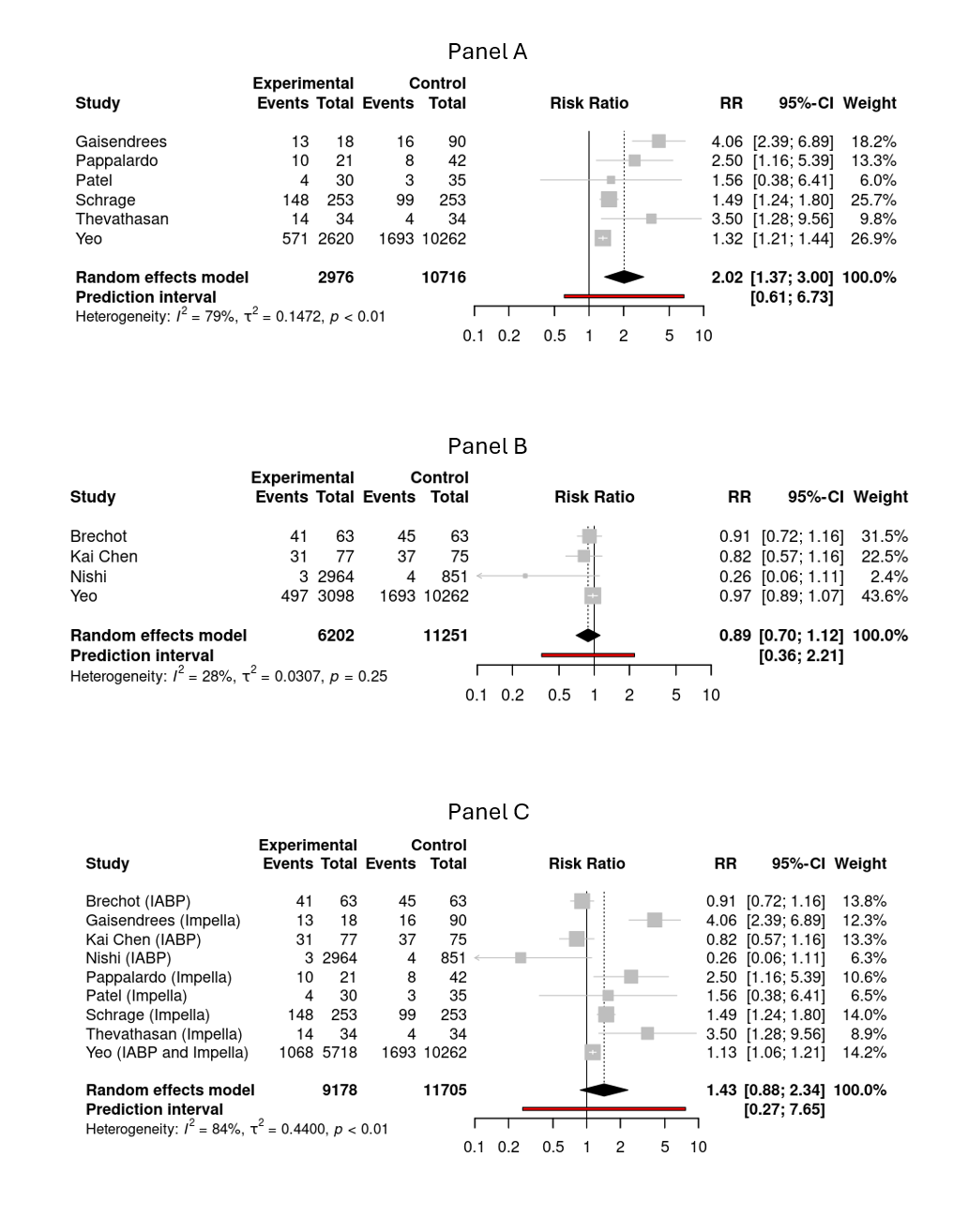


**Table S.2: Additional information to supplement Baseline Characteristics**

| **Study** | **Arm** | **Country** | **Length of Stay (days)** | **Time of Recording Mortality** |
| --- | --- | --- | --- | --- |
| Akanni | Impella | USA | 28 | IHM and 30-Day |
|  | No Unloading |  | 29 |  |
| Ayoma | IABP | Japan | - | IHM |
|  | No Unloading |  | - |  |
| Aso | IABP | Japan | - | IHM, 30-Day |
|  | No Unloading |  | - |  |
| Bjornsdottir | IABP | European and Arab countries | 19 | IHM |
|  | No Unloading |  | 21 |  |
| Brechot | IABP | - | 9 | IHM |
|  | No Unloading |  | 11 |  |
| Char | Impella | USA | - | IHM |
|  | IABP |  | - |  |
|  | No Unloading |  | - |  |
| Doll | IABP | - | - | IHM |
|  | No Unloading |  | - |  |
| Gaisendrees | Impella | Germany | 14 | IHM |
|  | No Unloading |  | 1 |  |
| Kai Chen | IABP | China | - | IHM |
|  | No Unloading |  | - |  |
| Kida | IABP | Japan | - | 30-Day |
|  | No Unloading |  | - |  |
| Kieserman | Impella | - | - | IHM, 6 Months |
|  | No Unloading |  | - |  |
| Lin | IABP | Taiwan | - | 2 Weeks |
|  | No Unloading |  | - |  |
| Nishi | IABP | Japan | - | IHM |
|  | No Unloading |  | - |  |
| Overtchouk | IABP | - | - | 30-Day |
|  | No Unloading |  | - |  |
| Pappalardo | Impella | Italy, Germany | - | IHM |
|  | No Unloading |  | - |  |
| Park | IABP | South Korea | - | IHM |
|  | No Unloading |  | - |  |
| Patel | Impella | USA | 6 | 30-Day, 1 Year |
|  | No Unloading |  | 6 |  |
| Ro | IABP | South Korea | - | IHM, 17 Months |
|  | No Unloading |  | - |  |
| Sakamoto | IABP | Japan | - | IHM |
|  | No Unloading |  | - |  |
| Schrage | Impella | - | - | 30-Day |
|  | No Unloading |  | - |  |
| Schmack | RUPV | Germany | 54 | 30-Day, 6 Months, 1 Year |
|  | No Unloading |  | 29 |  |
| Tepper | IABP | USA | - | IHM, 30-Day |
|  | No Unloading |  | - |  |
| Thevathasan | Impella | Germany | 7.5 | IHM |
|  | No Unloading |  | 5 |  |
| Van Den Brink | IABP | Europe | 34 | IHM, 1 Year |
|  | No Unloading |  | 27 |  |
| Wang | IABP | China | - | IHM |
|  | No Unloading |  | - |  |
| Yeo | IABP | - | 18 | IHM |
|  | Impella |  | 14 |  |
|  | No Unloading |  | 16 |  |
